# Supplementary material for: TACE and conformal radiotherapy vs. TACE alone for hepatocellular carcinoma: A randomised controlled trial
Source: JHEP Rep. 2023 Jan 29;5(4):100689. doi: 10.1016/j.jhepr.2023.100689 (PMC10017427; doi:10.1016/j.jhepr.2023.100689)
Supplement: Multimedia component 1 [file mmc1.pdf]

TACE and conformal radiotherapy *vs*. TACE alone for hepatocellular carcinoma: a randomised controlled trial

Cyrille Féray, Loic Campion, Philippe Mathurin, Isabelle Archambreaud, Xavier Mirabel, Jean Pierre Bronowicki, Emmanuel Rio, Christophe Perret, Laurent Mineur, Frederic Oberti, Yann Touchefeu, Jérôme Gournay, Hélène Regnault, Julien Edeline, Agnès Rode, Patrick Hillion, Jean Fréderic Blanc, Eric Nguyen Khac, Daniel Azoulay, Alain Luciani, Athena Galetto Preglisasco, Elodie Faurel-Paul, Hélène Auble, Françoise Mornex, Philippe Merle

Table of content

Fig. S1 2

Table S1 3

Table S2 6

Fig. S1. Liver PFS in 96 patients with centralized review of imaging blinded to therapeutical arm.

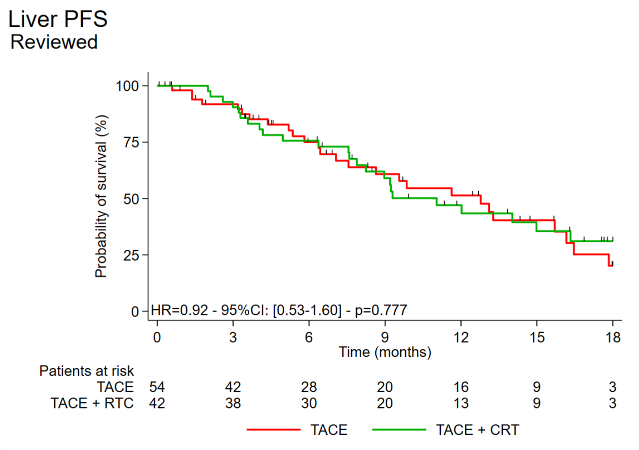

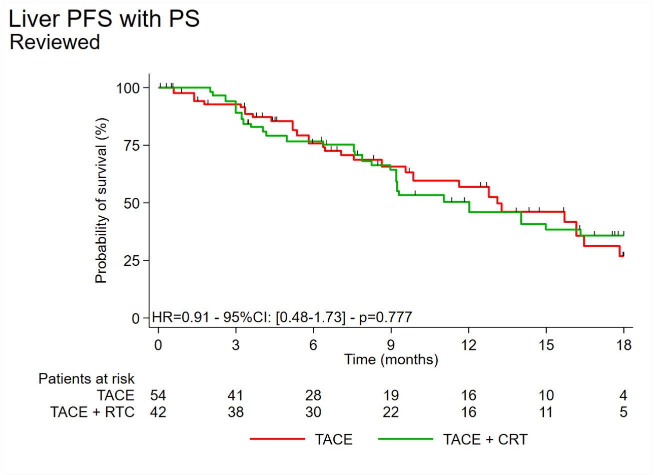

Table S1. Per-protocol patients characteristics

Variable	Global	TACE	TACE+RTC	*p*
**Gender**	100	55	45	*p = 0.8881*
Male	85 (85.0%)	47 (85.5%)	38 (84.4%)	
Female	15 (15.0%)	8 (14.5%)	7 (15.6%)	
**Age (years)**	100	55	45	*p = 0.1132*
Mean ± SD	70.7 ± 9.5	72.1 ± 9.6	69 ± 9.2	
Range	(48 ; 91)	(50 ; 91)	(48 ; 89)	
**Child-Pugh Score**	100	55	45	*p = 0.4919*
A	87 (87.0%)	49 (89.1%)	38 (84.4%)	
B	13 (13.0%)	6 (10.9%)	7 (15.6%)	
**ECOG**	100	55	45	*p = 0.1435*
ECOG 0	61 (61.0%)	30 (54.5%)	31 (68.9%)	
ECOG 1	39 (39.0%)	25 (45.5%)	14 (31.1%)	
**BCLC**	100	55	45	*p = 0.8984*
A	76 (76.0%)	42 (76.4%)	34 (75.6%)	
B	23 (23.0%)	12 (21.8%)	11 (24.4%)	
C	1 (1.0%)	1 (1.8%)	0 (0.0%)	
**ALBI Score**	100	55	45	*p = 0.0603*
Mean ± SD	-2.5 ± 0.5	-2.6 ± 0.5	-2.4 ± 0.4	
Range	(-3.5 ; -1.4)	(-3.5 ; -1.4)	(-3.2 ; -1.5)	
**ALBI Grade**	100	55	45	*p = 0.0421*
Grade 1	42 (42.0%)	28 (50.9%)	14 (31.1%)	
Grade 2	57 (57.0%)	26 (47.3%)	31 (68.9%)	
Grade 3	1 (1.0%)	1 (1.8%)	0 (0.0%)	
**Ethnic group**	100	55	45	*p = 0.5867*
Caucasian	97 (97.0%)	54 (98.2%)	43 (95.6%)	
Non-Caucasian	3 (3.0%)	1 (1.8%)	2 (4.4%)	
**Cirrhosis**	100	55	45	*p = 0.0212*
No	10 (10.0%)	9 (16.4%)	1 (2.2%)	
Yes	90 (90.0%)	46 (83.6%)	44 (97.8%)	
**Cause of liver disease**	100	55	45	*p = 0.2376*
Alcohol	52 (52.0%)	25 (45.5%)	27 (60.0%)	
Hepatitis C	10 (10.0%)	5 (9.1%)	5 (11.1%)	
Hepatitis B	2 (2.0%)	1 (1.8%)	1 (2.2%)	
Alcohol+Metabolic	10 (10.0%)	5 (9.1%)	5 (11.1%)	
Alcohol+Hepatitis C	2 (2.0%)	2 (3.6%)	0 (0.0%)	
Hemochromatosis+Metabolic	1 (1.0%)	0 (0.0%)	1 (2.2%)	
Hemochromatosis	4 (4.0%)	3 (5.5%)	1 (2.2%)	
Autoimmune	1 (1.0%)	0 (0.0%)	1 (2.2%)	
Metabolic	7 (7.0%)	4 (7.3%)	3 (6.7%)	
Other	1 (1.0%)	1 (1.8%)	0 (0.0%)	
Normal liver	10 (10.0%)	9 (16.4%)	1 (2.2%)	
**Diagnostic CHC**	100	55	45	*p = 0.7960*
Radiology	68 (68.0%)	38 (69.1%)	30 (66.7%)	
Biopsy	32 (32.0%)	17 (30.9%)	15 (33.3%)	
**Interval between diagnosis and randomization (months)**	100	55	45	*p = 0.2528*
Mean ± SD	10.8 ± 18.4	12.7 ± 21.9	8.4 ± 12.8	
Range	(0.3 ; 98.7)	(0.3 ; 98.7)	(0.6 ; 49.4)	
**Previous therapy**	100	55	45	*p = 0.5870*
None	74 (74.0%)	39 (70.9%)	35 (77.8%)	

Curative	14 (14.0%)	8 (14.5%)	6 (13.3%)	
Curative+TACE	3 (3.0%)	3 (5.5%)	0 (0.0%)	
Palliative	9 (9.0%)	5 (9.1%)	4 (8.9%)	
**Previous curative therapy**	17	11	6	*p = 0.1250*
Radiofrequency	11 (64.7%)	9 (81.8%)	2 (42.8%)	
Surgical Resection	3 (17.6%)	1 (9.1%)	2 (28.6%)	
Resection+Thermoablation	2 (11.8%)	1 (9.1%)	1 (14.3%)	
Alcoholization	1 (5.9%)	0 (0.0%)	1 (14.3%)	
**Previous palliative therapy**	12	8	4	*p = 0.0670*
TACE Lipiodol	8 (66.8%)	7 (87.5%)	1 (25.0%)	
TACE DC beads	1 (8.3%)	0 (0.0%)	1 (25.0%)	
TACE lipiodol+DC beads	1 (8.3%)	0 (0.0%)	1 (25.0%)	
TACE lipiodol+Sorafenib	1 (8.3%)	1 (12.5%)	0 (0.00%)	
Sorafenib	1 (8.3%)	0 (0.00%)	1 (25.0%)	
**Sorafenid**	100	55	45	*p = 1.0000*
No	98 (98.0%)	54 (98.2%)	44 (97.8%)	
Yes	2 (2.0%)	1 (1.8%)	1 (2.2%)	
**No. of lesions from radiology**	100	55	45	*p = 0.9536*
1	65 (65.0%)	36 (65.5%)	29 (64.4%)	
2	26 (26.0%)	14 (25.5%)	12 (26.7%)	
3	7 (7.0%)	4 (7.3%)	3 (6.7%)	
4	1 (1.0%)	0 (0.0%)	1 (2.2%)	
6	1 (1.0%)	1 (1.8%)	0 (0.0%)	
**Ascites (radiological)**	100	55	45	*p = 0.0743*
No	91 (91.0%)	53 (96.4%)	38 (84.4%)	
Yes	9 (9.0%)	2 (3.6%)	7 (15.6%)	
**Segmental Portal invasion (macroscopic)**	100	55	45	*p = 1.0000*
No	99 (99.0%)	54 (98.2%)	45 (100.0%)	
Yes	1 (1.0%)	1 (1.8%)	0 (0.0%)	
**Sum of tumor diameters (mm)**	99	54	45	*p = 0.8482*
Mean ± SD	54.3 ± 23.5	53.9 ± 23.1	54.8 ± 24.2	
Range	(11 ; 117)	(11 ; 113)	(17 ; 117)	
**TACE technique**	100	55	45	*p = 0.5617*
DC-Beads	75 (75.0%)	40 (72.7%)	35 (77.8%)	
Chimioembolisation lipiodol	25 (25.0%)	15 (27.3%)	10 (22.2%)	
**Hemoglobin (g/L)**	97	54	43	*p = 0.6970*
Mean ± SD	13.5 ± 1.7	13.4 ± 1.9	13.5 ± 1.5	
Range	(6 ; 17)	(6 ; 16.5)	(10.8 ; 17)	
**Leukocytes (103/mm3)**	97	54	43	*p = 0.8744*
Mean ± SD	5.8 ± 2.1	5.8 ± 2.2	5.8 ± 2.1	
Range	(2 ; 11.4)	(2 ; 10.3)	(2.2 ; 11.4)	
**Neutrophils (103/mm3)**	97	54	43	*p = 0.3204*
Mean ± SD	3.6 ± 1.6	3.8 ± 1.7	3.5 ± 1.5	
Range	(1.1 ; 8.4)	(1.2 ; 7.7)	(1.1 ; 8.4)	
**Lymphocytes (/mm3)**	100	55	45	*p = 0.2421*
Mean ± SD	1380.4 ± 648.2	1311.5 ± 556.3	1464.6 ± 743.2	
Range	(270 ; 4220)	(270 ; 3500)	(530 ; 4220)	
**Platelets (103/mm3)**	100	55	45	*p = 0.8633*
Mean ± SD	145.8 ± 76.8	144.5 ± 79.2	147.2 ± 74.5	
Range	(16 ; 365)	(16 ; 352)	(45 ; 365)	
**ASAT (IU/l)**	100	55	45	*p = 0.6255*
Mean ± SD	47.5 ± 28.1	46.2 ± 27.2	49 ± 29.3	
Range	(17 ; 160.8)	(18 ; 132.6)	(17 ; 160.8)	
**ALAT (IU/l)**	100	55	45	*p = 0.7005*
Mean ± SD	37.8 ± 28.5	36.8 ± 23.4	39.1 ± 33.9	
Range	(11 ; 208.2)	(11 ; 147)	(13.8 ; 208.2)	
**Alkaline Phosphatases (IU/l)**	99	55	44	*p = 0.7770*
Mean ± SD	125.4 ± 101.1	122.9 ± 90.5	128.7 ± 114	

Range	(56 ; 832)	(56 ; 693)	(62 ; 832)	
**Gamma-GT (IU/l)**	99	55	44	*p = 0.4498*
Mean ± SD	254.6 ± 260.6	236.8 ± 213.2	276.9 ± 311.1	
Range	(21 ; 1539)	(21 ; 1108)	(37 1539)	
**Total bilirubin (mG/L)**	100	55	45	*p = 0.2574*
Mean ± SD	17.2 ± 9.8	16.2 ± 9.1	18.4 ± 10.6	
Range	(4 ; 50)	(4 ; 44.5)	(5 ; 50)	
**Alfa FP (ng/mL)**	100	55	45	*p = 0.1638*
Mean ± SD	179.8 ± 635.8	99.5 ± 321.6	277.9 ± 874.4	
Range	(0.3 ; 4290)	(0.5 ; 1960.6)	(0.3 ; 4290)	
**Alfa FP (ng/mL)**	100	55	45	*p = 0.1178*
Low (<200)	86 (86.0%)	50 (90.9%)	36 (80.0%)	
High (>=200)	14 (14.0%)	5 (9.1%)	9 (20.0%)	
**INR**	100	55	45	*p = 0.9060*
Mean ± SD	1.2 ± 0.4	1.3 ± 0.5	1.2 ± 0.4	
Range	(0.9 ; 3.3)	(1 ; 3.3)	(0.9 ; 3.2)	
**Albumin (G/L)**	100	55	45	*p = 0.0842*
Mean ± SD	38.2 ± 4.8	39 ± 4.8	37.3 ± 4.7	
Range	(26 ; 49.1)	(28.7 ; 49.1)	(26 ; 45)	
**Creatinine (µmol/L)**	99	55	44	*p = 0.6251*
Mean ± SD	87 ± 59.7	84.3 ± 47.6	90.3 ± 72.4	
Range	(42 ; 524)	(44.3 ; 399)	(42 ; 524)	
**Sodium (mmol/L)**	98	54	44	*p = 0.5537*
Mean ± SD	138.9 ± 2.9	138.7 ± 3.1	139 ± 2.7	
Range	(128 ; 145)	(128 ; 145)	(131 ; 145)	
**Lymphocytes (/mm3)**	100	55	45	*p = 0.3223*
>=800	88 (88.0%)	50 (90.9%)	38 (84.4%)	
<800	12 (12.0%)	5 (9.1%)	7 (15.6%)	
**Platelets (/mm3)**	100	55	45	*p = 0.0214*
>=75000	87 (87.0%)	44 (80.0%)	43 (95.6%)	
<75000	13 (13.0%)	11 (20.0%)	2 (4.4%)	
**Total bilirubin (mg/L)**	100	55	45	*p = 0.1913*
<20	71 (71.0%)	42 (76.4%)	29 (64.4%)	
>=20	29 (29.0%)	13 (23.6%)	16 (35.6%)	

Table S2

Concordance between blinded and non-blinded review

	Non-blinded review					
Blinded revi	Censored	Stable disease	Progression	Complete response	Partial response	Total
Complete response	1	0	6	10	1	18
Partial resp	0	5	9	1	8	23
Stable disea	1	2	3	0	5	11
Progression	0	5	23	0	2	30
Censored	12	0	0	2	0	14
Total	14	12	41	13	16	96

Among the 96 patients with both evaluations, 16 were censored due to a very short follow-up period (only evaluation at inclusion available).

For the 80 patients for whom both evaluations were available:

	Non-blinded review		
Blinded revi	No progression	Progression	Total
No progressi	32	18	50
Progression	7	23	30
Total	39	41	80

Agreement = 68.75%/Expected agreement=49.69%/**Kappa=0.3789** (p=0.0002)
