# Supplementary material for: TACE and conformal radiotherapy vs. TACE alone for hepatocellular carcinoma: A randomised controlled trial
Source: JHEP Rep. 2023 Jan 29;5(4):100689. doi: 10.1016/j.jhepr.2023.100689 (PMC10017427; doi:10.1016/j.jhepr.2023.100689)
Supplement: Multimedia component 2 [file mmc2.docx]

Protocole TACERTE

Numéro ID RCB : 2010-A01089-30

Ref BRD : 10/6-M

EFFECTIVENESS OF THE COMBINATION OF HIGH-DOSE CONFORMATIONAL RADIOTHERAPY AND HEPATIC INTRA-ARTERIAL CHEMO-EMBOLIZATION IN THE TREATMENT OF HEPATOCELLULAR CARCINOMA

| Coordinating investigator:  Professeur FERAY Cyrille  PUPH Hépatologue  Service d’hépato gastro-entérologie  CHU Nantes  9 quai Moncousu, Nantes  Tél : 0240083152  cyrille.feray@chu-nantes.fr |
| --- |
|  |
| Methodologist    Dr Loïc Campion  CRLCC Gauducheau  Bd Jacques Monod  44093 St Herblain  Tél : 0240679900  l-campion@nantes.fnclcc.fr |

| Promoter :  CHU Hotel Dieu  1 place Alexis Ricordeau  44093 Nantes Cedex |
| --- |

| Cancéropôle de référence :  Cancéropôle grand-ouest |
| --- |

# SOMMAIRE

[SOMMAIRE 2](#_Toc276466036)

[1. Informations générales comprenant notamment : 5](#_Toc276466037)

[PAGE DE SIGNATURE 6](#_Toc276466038)

[LISTE DES ABREVIATIONS 7](#_Toc276466039)

[RESUME 8](#_Toc276466040)

[2. Justification scientifique et description générale de la recherche 11](#_Toc276466041)

[2.1. Problème de santé publique 11](#_Toc276466042)

[2.2 Positionnement des travaux dans le contexte des connaissances actuelles 11](#_Toc276466043)

[2.2.1. Traitements "physiques" du carcinome hépatocellulaire 11](#_Toc276466044)

[La transplantation hépatique (TH) 11](#_Toc276466045)

[La résection 11](#_Toc276466046)

[La radiofréquence ablative (RFA) 12](#_Toc276466047)

[Thérapies par cathétérisme de l'artère hépatique (TACE) 12](#_Toc276466048)

[2.2.2 Chimiothérapie du CHC 12](#_Toc276466049)

[2.2.3 Problèmes spécifiques des essais thérapeutiques sur le CHC [9] 12](#_Toc276466050)

[La survie comme critère 12](#_Toc276466051)

[Traitements combinés 13](#_Toc276466052)

[Critères RECIST adaptés et Critères OMS EASL 13](#_Toc276466053)

[Evaluation de la Qualité de Vie 13](#_Toc276466054)

[Étude médico-économique 14](#_Toc276466055)

[2.3 Dénomination et description des thérapies mises en œuvre 14](#_Toc276466056)

[2.3.1 TACE DC Beads 14](#_Toc276466057)

[2.3.2 La radiothérapie externe conformationnelle (RTC) 15](#_Toc276466058)

[TDM dosimétrique 15](#_Toc276466059)

[Délinéation des volumes 16](#_Toc276466060)

[Technique d’irradiation 16](#_Toc276466061)

[Histogrammes-dose-volume 16](#_Toc276466062)

[2.4. Résumé des résultats des essais non cliniques et des essais cliniques pertinents au regard de la recherche biomédicale concernée 17](#_Toc276466063)

[2.5. Bénéfices et risques pour les personnes se prêtant à la recherche 18](#_Toc276466064)

[2.5.1. Bénéfices 18](#_Toc276466065)

[Bénéfices individuels 18](#_Toc276466066)

[Bénéfices collectifs 18](#_Toc276466067)

[2.5.2. Risques 18](#_Toc276466068)

[TACE par DC Beads 18](#_Toc276466069)

[Radiothérapie externe conformationnelle 18](#_Toc276466070)

[Risques et contraintes psychologiques 19](#_Toc276466071)

[Contraintes liées à la recherche 19](#_Toc276466072)

[Evaluation de la toxicité aiguë 19](#_Toc276466073)

[RTOG/EORTC Score de morbidité tardive de la radiothérapie 19](#_Toc276466074)

[3. Objectifs de la recherche et critères de jugements 20](#_Toc276466075)

[3.1. Objectif de la recherche 20](#_Toc276466076)

[*3.1.1.* *Objectif principal* 20](#_Toc276466077)

[3.1.2. Objectifs secondaires 20](#_Toc276466078)

[3.2. Enoncé du critère d'évaluation principal et des critères d'évaluation secondaires 20](#_Toc276466079)

[3.2.1. Critère de jugement principal 20](#_Toc276466080)

[3.2.2. Critères de jugement secondaires 21](#_Toc276466081)

[4. Design de la recherche 22](#_Toc276466082)

[4.1. Méthodologie générale de la recherche 22](#_Toc276466083)

[4.1.1. Plan expérimental 22](#_Toc276466084)

[4.1.2. Déroulement de l’étude 22](#_Toc276466085)

[4.1.3. Etude médico-économique 22](#_Toc276466086)

[4.1.3.1. Type des coûts pris en compte 22](#_Toc276466087)

[1. Coûts directs médicaux 22](#_Toc276466088)

[2. Coûts directs non médicaux 23](#_Toc276466089)

[4.1.3.2 . Spécifications concernant le calcul des coûts 23](#_Toc276466090)

[4.2. Schéma général de l’étude 24](#_Toc276466091)

[5. Population étudiée 24](#_Toc276466092)

[5. Population étudiée 25](#_Toc276466093)

[5.1. Description de la population à étudier 25](#_Toc276466094)

[5.1.1. Description de la population 25](#_Toc276466095)

[5.1.2. Description et nombre de sujets prévus pour l’échantillon 25](#_Toc276466096)

[5.1.3. Modalités de recrutement 25](#_Toc276466097)

[5.2. Critères d'inclusion 25](#_Toc276466098)

[5.3. Critères de non-inclusion 26](#_Toc276466099)

[6. Traitements utilisés pendant l’étude 27](#_Toc276466100)

[6.1. Description du ou des traitements nécessaires à la réalisation de la recherche 27](#_Toc276466101)

[6.1.1. Pour la TACE 27](#_Toc276466102)

[6.1.2. Pour la RTC 27](#_Toc276466103)

[6.2. Traitements autorisés 27](#_Toc276466104)

[6.3. Traitements interdits 27](#_Toc276466105)

[6.4. Méthodes de suivi de l’observance au traitement 27](#_Toc276466106)

[6.5. Conditions de stockage des médicaments expérimentaux 27](#_Toc276466107)

[6.5.1. Description du stockage à la pharmacie 27](#_Toc276466108)

[6.5.2. Description du stockage dans le service 28](#_Toc276466109)

[7. Déroulement de l’étude 29](#_Toc276466110)

[7.1. Techniques d’études et d’analyses 29](#_Toc276466111)

[7.1.1. Description des mesures prises pour réduire et éviter les biais incluant notamment 29](#_Toc276466112)

[7.1.1.1. Le tirage au sort 29](#_Toc276466113)

[7.1.1.2. Les méthodes de mise en insu 29](#_Toc276466114)

[7.1.1.3. Dispositions mises en œuvre en vue du maintien de l'insu et procédures de levée de l'insu, le cas échéant 29](#_Toc276466115)

[7.2. Calendrier de l’étude 29](#_Toc276466116)

[Flow-chart de l’étude TACERTE 35](#_Toc276466117)

[7.3. Identification de toutes les données à recueillir directement dans les cahiers d'observation, qui seront considérées comme des données sources 36](#_Toc276466118)

[7.4. Règles d’arrêt de la participation d’une personne 36](#_Toc276466119)

[7.4.1 Critères d'arrêt prématuré du traitement ou d'exclusion d'une personne de la recherche 36](#_Toc276466120)

[7.4.2. Modalités d’arrêt prématuré du traitement ou d’exclusion d’une personne de la recherche 36](#_Toc276466121)

[7.4.3. Critères d’arrêt de la recherche 37](#_Toc276466122)

[8. Data management et statistiques 38](#_Toc276466123)

[8.1. Recueil et traitement des données de l’étude 38](#_Toc276466124)

[8.1.1. Recueil des données 38](#_Toc276466125)

[8.1.2. Traitement des données 39](#_Toc276466126)

[8.1.3. Archivage des données 39](#_Toc276466127)

[8.2. Statistiques 39](#_Toc276466128)

[8.2.1. Description des méthodes 40](#_Toc276466129)

[Variables prises en compte 40](#_Toc276466130)

[Tests statistiques utilisés 40](#_Toc276466131)

[Analyses intermédiaires prévues 41](#_Toc276466132)

[8.2.2. Justification statistique du nombre d’inclusion 41](#_Toc276466133)

[8.2.3. Degré de signification statistique prévu 41](#_Toc276466134)

[8.2.4. Critères statistiques d'arrêt de la recherche 41](#_Toc276466135)

[8.2.5. Méthode de prise en compte des données manquantes, inutilisées ou non valides 41](#_Toc276466136)

[8.2.6. Choix des personnes à inclure dans les analyses 42](#_Toc276466137)

[8.2.7. Randomisation 42](#_Toc276466138)

[9. Vigilance et gestion des événements indésirables 43](#_Toc276466139)

[9.1. Définitions 43](#_Toc276466140)

[9.1.1. Evènements indésirables (EvI) 43](#_Toc276466141)

[9.1.2. Effets Indésirables (EI) 43](#_Toc276466142)

[9.1.3. Evènements ou effets indésirables graves EVIG/EIG 44](#_Toc276466143)

[9.1.4. Effets ou évènements indésirables attendus 45](#_Toc276466144)

[9.1.5. Effets indésirables graves inattendus 45](#_Toc276466145)

[9.2. Paramètres d'évaluation de la sécurité 45](#_Toc276466146)

[9.2.1. Critères d’évaluation particuliers liés à la sécurité 45](#_Toc276466147)

[9.2.2. Méthodes et calendrier prévus pour mesurer, recueillir et analyser les paramètres d'évaluation de la sécurité 45](#_Toc276466148)

[9.3. Liste des EI attendus 46](#_Toc276466149)

[9.3.1 Concernant la radiothérapie 46](#_Toc276466150)

[9.3.2. Concernant la Chimioembolisation par DC Beads 46](#_Toc276466151)

[9.4. Gestion des évènements indésirables 47](#_Toc276466152)

[9.4.1. Notification des EIG 47](#_Toc276466153)

[9.4.2. Déclaration aux autorités compétentes 48](#_Toc276466154)

[9.4.3 Comité de surveillance indépendant 48](#_Toc276466155)

[9.4.4. Rapport annuel de sécurité (RAS) 49](#_Toc276466156)

[9.5. Modalités et durée du suivi des personnes suite à la survenue d'événements indésirables 49](#_Toc276466157)

[10. Aspects administratifs et réglementaires 50](#_Toc276466158)

[10.1. Droit d'accès aux données et documents source 50](#_Toc276466159)

[10.2. Monitoring de l’essai – Risque D (100%) 50](#_Toc276466160)

[10.3. Inspection / Audit 51](#_Toc276466161)

[10.4. Considérations éthiques 51](#_Toc276466162)

[10.4.1. Consentement éclairé écrit 51](#_Toc276466163)

[10.4.2. Comité de Protection des Personnes 51](#_Toc276466164)

[10.4.3. Anonymat des patients 51](#_Toc276466165)

[10.4.4. Données informatisées 52](#_Toc276466166)

[10.5. Amendements au protocole 52](#_Toc276466167)

[10.6. Déclaration aux autorités compétentes 52](#_Toc276466168)

[10.7. Financement et assurance 52](#_Toc276466169)

[10.8. Règles relatives à la publication 53](#_Toc276466170)

[11. Faisabilité du projet 54](#_Toc276466171)

[11.1 Effectifs 54](#_Toc276466172)

[11.2 Possibilité de recrutement 54](#_Toc276466173)

[11.3 Concurrence des autres essais 54](#_Toc276466174)

[11.4. Organisation de l’essai et management 54](#_Toc276466175)

[11.4.1. Coordination du projet 54](#_Toc276466176)

[11.4.2. Dans chaque centre 55](#_Toc276466177)

[Liste des annexes 56](#_Toc276466178)

# 1. General information:

EFFECTIVENESS OF THE COMBINATION OF HIGH-DOSE CONFORMATIONAL RADIOTHERAPY AND HEPATIC INTRA-ARTERIAL CHEMO-EMBOLIZATION IN THE TREATMENT OF HEPATOCELLULAR CARCINOMA

- - **N° ID RCB :** 2010-A01089-30
  - Référence CPP :
  - N° interne BRD : 10/6-M

- The name and contact details of the promoter: CHU Hôtel Dieu, 1 place Alexis Ricordeau, 44093 Nantes Cedex

- - **-Managing the project:** Cellule de promotion de la recherche clinique, Immeuble Deurbroucq, 5 allée de l’île Gloriette, 44093 Nantes Cedex. Tél : 02 53 48 28 56– Fax : 02 53 48 28 36

E-mail : [valérie.collet-poirier@chu-nantes.fr](mailto:anne.omnes@chu-nantes.fr)

- Persons authorized to sign the protocol and its possible modifications on behalf of the sponsor: Pr Cyrille FERAY Investigator Coordinator

- Contact details of the research manager at sponsor level: Ms Anne OMNES Head of the Clinical Research Promotion Unit, DAMR, CHU Nantes

- Coordinating investigator: Pr Cyrille FERAY, Hepato-gastroenterology department, CHU Nantes

- Deputy coordinator: Pr Philippe MERLE

- Methodologist: Dr Loïc CAMPION, CRLCC René Gauducheau, Saint-Herblain

- Principal Investigators: see list in appendix 1

- Composition of the independent supervisory committee: see list in appendix 16SIGNING PAGE

This page is a protocol validation page. It is used to certify the accuracy of the protocol data.

It must include the following signatures:

| Nom | Coordonnées | Date | Signature |
| --- | --- | --- | --- |
| Investigateur coordonnateur | Pr FERAY Cyrille  PUPH Hépatologue  Service d’hépato gastro-entérologie  CHU Nantes  9 quai Moncousu, Nantes  Tél : 02 40 08 31 52  [cyrille.feray@chu-nantes.fr](mailto:cyrille.feray@chu-nantes.fr) |  |  |
| Promoteur | OMNES Anne  CHU Hôtel Dieu  1 place Alexis Ricordeau  44093 Nantes Cedex |  |  |

# LISTE DES ABREVIATIONS

ARC: Clinical Research Associate

HCC: Hepatocellular carcinoma

CRF: Case Report Form (observation book)

EASL: European Association for the Study of the Liver

ECG: Electrocardiogram

NFS: Complete blood count

OAR: Organs at risk

RECIST: Response Evaluation Criteria In Solid Tumor

RFA: Ablative Radiofrequency

RFC: Conformal External Radiation Therapy

RILD: Radiation Induced Liver Disease

TACE: Hepatic Artery Catheterization Therapy – Chemoembolization

CT: Computed tomography – Scanner

TH: Liver transplant

HBV: Hepatitis B virus

HCV: Hepatitis C virus

# RESUME

| Coordinating center:  Nantes University Hospital  Coordinating investigator: Pr Cyrille Feray | | Inclusion period: 24 months  Follow-up period: 18 months for the evaluation of the primary endpoint  Overall duration of the trial: 42 months |
| --- | --- | --- |
| Study title | Efficacy of the combination of high-dose conformal radiotherapy (CRT) and hepatic intra-arterial chemoembolization using DC Beads (TACE DC Beads) in the treatment of hepatocellular carcinoma (HCC) | |
| Objectives | Main: To obtain a survival without hepatic tumor progression in group 1 (TACE DC Beads followed by a RTC) superior by 20% compared to group 2 (2 TACE DC Beads minimum 8 weeks apart) (reference treatment) on the hepatic tumor progression-free survival at 18 months**.**  Secondary: Assess:  - acute toxicity (within 90 days of treatment)  - late toxicity (after 90 days of treatment)  - quality of life until disease progression or until death  - local control rate  - overall survival  - the economic superiority of the TACE-RTC combination compared to the reference treatment | |
| Methodology | Randomised, controlled, phase II, multicentre trial comparing the benefit of CRT after treatment with a course of TACE DC Beads versus treatment with a minimum of 2 courses of TACE DC Beads (reference treatment). | |
| Therapeutic modalities | For the TACE DC Beads:  4ml of DC Beads (1 dose with 300-500 µm microspheres then with 500-700 µm microspheres) loaded with 150mg of doxorubicin.  Group 1: 1 TACE session  Group 2: 2-3 TACE sessions spaced 2 months apart  For radiotherapy:  54 gy in about 18 sessions for 3-4 weeks | |
| Study population | Patient with HCC who cannot benefit from resection, ablative radiofrequency (AFR) or an immediate indication for liver transplantation, having an indication for treatment with TACE DC Beads and who can technically undergo HCC irradiation. | |
| Number of patients | Number of patients: 174 included  Divided into 2 treatment groups according to the 1:1 ratio:  - group 1: 87 patients under TACE-RTC treatment   - - group 2: 87 patients under TACE treatment (reference treatment) | |

| Efficacy criteria | Primary endpoint:  The primary endpoint is the tumor progression-free survival time defined radiologically on the EASL (European Association of the Study of the Liver) criteria.  In the context of this trial, the objective response includes the rate of complete responses and partial responses. The response criteria are defined on the change in diameter of the total viable volume.  At the end of the evaluation of the response, the patient will be classified in one of the following 8 categories:  1- complete response (CR): disappearance of all viable target lesions  2- partial response (PR): reduction of at least 50%  3- stable disease (ST): decrease of less than 50% or increase of less than 25%  4- progression (PG): increase of more than 25% or appearance of new lesions  5- early death from neoplastic cause (DCD K)  6- early death from a toxic cause (DCD Tox)  7- early death from another cause (DCD X)  8- unknown (not evaluable, insufficient data) (I)  All patients who met the eligibility criteria should be included in the primary analysis of response rates. Groups 4.5 and 8 should be considered treatment failure.  Secondary criteria:  - Overall survival  - Rate of CR, PR and ST at 6, 12 and 18 months of follow-up  - Side effects  - Quality of life questionnaire  - Medico-economic study |
| --- | --- |
| Inclusion criteria | - - Age ≥ 18 years old - - ECOG 0-1 - - Life expectancy ≥ 6 months - - HCC proven histologically or according to radiological and biochemical criteria (EASL/AASLD) in cirrhotic patients - - Maximum lesion ≤ 9 cm - - Not eligible for surgery, percutaneous therapy - - Early Child-Pugh A or B (7 points for the Child-Pugh score) - - AST and ALT < 7 x ULN - - Technical possibility of conformal irradiation of the tumor mass - - Technical possibility of treatment with DC Beads - - The entire tumor mass must be able to receive the DC Beads - - Written informed consent signed by the patient |
| Non-inclusion criteria | - - Metastatic HCC disease - - Uncontrolled viral replication B - - Possibility of liver transplantation within 12 months - - History of abdominal radiotherapy (including lipiciocis or yttrium90) - - Lack of means or refusal to use effective means of contraception for men or women of childbearing age. - -Pregnancy and breast feeding - - Contraindication to TACE by DC Beads or RTC - - Any other concomitant experimental treatmentContre indication de la doxorubicine |
| Statistical methods | Statistical analyzes will be descriptive and comparative, all done with stratification on potential confounding parameters.  The comparisons of the various groups of interest will be made for numerical data by Student's t test or ANOVA and for discrete data by Pearson's Chi² test.  Survival curves will be plotted using the Kaplan-Meier method and comparisons of survival curves will use the logrank test. Multi-parametric analyzes for the search for independent prognostic factors for progression-free survival will be carried out using the Cox regression model with stratification.  The analyzes will be done by intention to treat and per protocol following a bilateral scheme. The p of significance limit will be set at 5%.  Analysis software will be SAS 9.2 (SAS Institute Inc., Cary, NC, USA) and Stata SE 10. |
| Important Notes | Patients may have had all previous therapies (except irradiation), all etiologies of liver disease, with or without HIV infections.  There are no restrictions on alcohol intake or age.  The perspective or registration in liver transplantation is possible.  Multifocal locations that are distant from each other can be irradiated. The feasibility of irradiation can only be judged by the radiotherapist. |

# 2. Scientific justification and general description of the research

## Public health issue

Hepatocellular carcinoma (HCC) is the 5th cancer in the world and the 3rd in terms of mortality [1] [2]. Its incidence is increasing in Western countries under the influence of the epidemic of viral hepatitis C (HCV) and B (HBV) but also by the emergence of patients with dysmetabolic cirrhosis.

There is a risk of a significant increase in HCC until 2020. In France, the annual incidence of HCC is currently 7000, most often occurring on advanced liver disease (due to HCV in 30% of cases). The optimal treatment, liver transplantation, is possible in a few hundred patients per year. Diagnosis is often late and conventional chemotherapies are not very active, especially since hepatocellular function is often impaired due to the underlying liver disease. For these reasons, the prognosis of HCC remains poor, with mortality close to its incidence.

## 2.2 Current therapies for HCC

### 2.2.1. "Physical" treatments for hepatocellular carcinoma

#### Liver transplantation (LT)

This is the best option as it eradicates the possibility of recurrence on the cirrhotic liver. It exposes to a risk of development of possible extra-hepatic metastases and is only feasible in a few hundred patients selected per year: those without metastases having a main tumor whose diameter is less than 7 cm and carrying a maximum of 3 locations. The waiting period is such that HCC treatment is systematically considered unless a related donor is willing [3]. TH is the only solution for those with liver failure. HT may also be indicated if the tumor mass fulfills the above-described criteria (known as Milan or UCSF) after therapy (down-staging or loss of the tumor mass) [4].

#### Liver resection

Resection of an HCC is only possible if hepatic function is correct with moderate portal hypertension and relatively low tumor mass. It is considered a potentially curative therapy and also affects a minority of patients.

#### Ablative radiofrequence (RFA)

It allows satisfactory treatment for small tumors with results similar to resection. It is less demanding on liver function and portal hypertension and can be considered potentially curative. It is aimed at uni or binodular forms [5].

#### Trans Arterial chemo embolization (TACE)

The exclusively arterial vascularization of CHCs allows endovascular approaches combining intra-tumor infusion of chemotherapeutic or radioactive substances (lipiocis®, Yttrium®) and embolization. The level of embolization can be macroscopic as in classic lipiodol chemoembolization or microscopic as in the new techniques using particles loaded with radioactive substance (Yttrium 90) or chemotherapeutic substance (DC Beads). Most patients recover if liver function is preserved (child <B8) from intra-arterial therapy.

2.2.2 Chemotherapy of HCC

After a long phase of negative results, chemotherapy is now dominated by sorafenib (Nexavar ®) [6] [7], a so-called targeted anti-angiogenic therapy that also inhibits pathways that are often activated in HCC. Sorafenib has a certain action on advanced forms of HCC. Its frequent side effects (asthenia, cutaneous, hemorrhagic disorders) and its cost limit its use. This product has marketing authorization if liver function is preserved and in the absence of curative therapy. Its use will therefore be extended to populations subjected to these therapies. Trials of the sorafenib + TACE combination are ongoing [8].

### 2.2.3 Specific issues of therapeutic trials in HCC [9]

#### Survival as a criterion

Either the patient cannot receive any known therapy and the trial of a new approach or molecule is simplified. Nevertheless, the survival of the patient depends on the evolution of the tumor but also on that of the hepatic function. This situation of competing risks characteristic of HCC increases the number of subjects to include when survival is the criterion..

Either the liver function is still preserved but no curative therapy can be considered at this stage because of too large a tumor mass. The patient is then likely to undergo TACE or CRT and/or receive sorafenib or enter trials like ours. The possibility of spontaneous hepatic decompensation or due to the therapies and vice versa, the possibility of a melting of the tumor mass (down-staging) authorizing curative therapies are the two factors which will influence the patient's survival. It is therefore added to the competition of risks (decompensation), the therapeutic competition offered by the down-staging or even by the evolution of targeted therapies. It is clear that survival-based therapeutic trials in this setting would require even more patients and prolonged follow-up. It is therefore more reasonable to take into account the criterion of efficacy on the tumor.

Combined therapies

In the field of HCC treatment, there is only one trial demonstrating the superiority of a combination of two validated HCC therapies over monotherapy. It is the adjuvant Lipiocis® after hepatic resection [10]. Another paper, that of Cheng [11] combining TACE and RFA, quickly proved to be a sham, the study published in JAMA being in fact retrospective and non-randomized [12]. In current practice, each therapeutic line (TACE, RFA, surgery, sorafenib) is tried after the failure of the previous one. Randomized, controlled trials are in progress combining sorafenib + validated therapy but few combining two physical therapies. An ongoing PHRC was testing the interest of TACE Lipiocis® as an adjuvant to radical therapy (RFA or resection) but lipiocis is no longer marketed due to pulmonary side effects.

#### Critères RECIST adaptés et Critères OMS EASL

Tumors can necrotize spontaneously and most often under the effect of therapies. Simple RECIST (Response Evaluation Criteria In Solid Tumor) measurements (see appendix 7) are insufficient. The current rule, inspired by the Barcelona school, is to take into account the evolution of the vascularized part after local or general treatment. It is therefore necessary to take into account in this study the EASL criteria (see appendix 6), which study the tumor devascularization rather than the size of the tumor alone..

Quality of Life Assessment

It will be done using the EORTC-QLQ C30 (version 3) (see appendix 9). This is a cancer-specific self-administered questionnaire [13]. Internal validation of this questionnaire, made up of 30 items, made it possible to identify 15 dimensions and calculate 15 scores: 5 functional ability scores (physical ability, ability to work or perform any household task, cognitive ability, emotional state , social status), a global quality of life score, a financial problems score and 8 symptom scores (fatigue, nausea/vomiting, pain, dyspnea, sleep disturbance, loss of appetite, constipation, diarrhea). The response modalities to the items vary from 4 to 7 on a Likert-type scale. If at least half of the items of each of the scores are filled in then the scores can be generated. Overall health and functional scores range from 0 (worse) to 100 (best) while symptom scores range from 100 (worse) to 0 (best). The average filling time is 10 minutes.

Medico-economic study

A cost study will be carried out. It will be limited from the point of view of the community, by limiting itself to the estimation of direct medical and non-medical costs, excluding indirect costs. These costs will be assessed prospectively, from the start of radiotherapy and over a period of one month following the end of treatment..

## 2.3 Name and description of the therapies used in the trial

### TACE DC Beads

A recent multicentre randomized trial involving 200 patients (PRECISION-V study) [14] prospectively and randomly compared these doxorubicin-loaded particles (DC Beads) during TACE with TACE using lipiodol. DC Beads tend to improve the local response and are significantly better tolerated from a hepatic and general point of view. They standardize and improve the old technique of lipodol TACE which will tend to disappear. They involve selective embolization, whereas lipodol techniques still frequently use global hepatic embolization..

DC Beads (Biocompatibles UK Ltd.) are a novel system for intra-arterial embolization of non-absorbable hydrogel microspheres loaded with doxorubicin. Preclinical [15] [16] and clinical [17] [18] studies point to good intra-tumor diffusion of chemotherapy, less hepatic and general toxicity and activity probably superior to techniques Lipiodolated TACE.

Patients will receive DC Beads (100-300 µm microspheres or possibly with 300-500 µm microspheres at the discretion of the radiologist (in the case of arteriovenous fistulas)) loaded with a maximum of 75 mg of doxorubicin per session (i.e. 4 mg of DC Beads and 150 mg of doxorubicin) diluted in 10 to 20 ml of anionic contrast product and without the addition of lipiodol.

The injection must be as selective as possible. It should be slow at 1 ml/min.

The microspheres will be delivered by catheterization of the hepatic artery and its branches in patients under light sedation without intubation, by hepatic arteriography in the segment or segments concerned. In both groups, a technical embolization failure (not all the nodules were injected) may lead to a second embolization. In all cases, the duration of hospitalization is a minimum of 3 days in the absence of complications..

Maximum devascularization is preferred in the TACE-RTC patient group so that all tumor nodules can benefit from the combination of the two therapies.

It is left to radiologists the possibility, depending on the habits of each center, of completing the embolization of patients with microbeads not charged with chemotherapy..

### 2.3.2 Conformal external radiotherapy (RTC)

Advances in imaging and computer technology have facilitated the delivery of conformal external radiotherapy in HCC. Although the accumulated experience of hepatic radiotherapy is limited, the technologies and tools required to deliver radiotherapy safely have been rapidly developed. Various doses and fractionations have been used to irradiate HCC, and radiotherapy has often been used in combination with other therapies, such as hepatic arterial chemoembolization. The results obtained with radiotherapy, exclusively or in combination with chemoembolization, seem better than those of exclusive chemoembolization, but randomized trials are needed [19].

Dosimetric CT

It is advisable to perform two dosimetric scans. A first CT scan without injection of contrast product to characterize the chemoembolized nodule(s), followed by a CT scan with injection of iodinated contrast product with acquisition of images in arterial time to include in the macroscopic tumor volume (GTV), any peripheral areas taking up the contrast.

If it is impossible to perform two CT scans, only the CT scan with injection and acquisition of images in arterial time is mandatory.

The examination is performed in the supine position, arms raised, with or without block under the legs.

Recommendation: the patient is immobilized by a technique chosen by the center: respiratory servoing and/or abdominal compression with or without a vacuum mattress.

The cutting planes are made in contiguous sections with a maximum thickness of 3 mm, including the tumor volume and all the organs at risk (OAR). In order to obtain good quality images, the scanner is performed by immobilizing the target volume (CTV) in the patient as much as possible: either in apnea (respiratory control), or with abdominal compression, or in free breathing if the scanner manages 4D (scan reconstruction corresponding to a selected phase of the respiratory cycle).

### Tumoral volumes

The macroscopic tumor volume (GTV) corresponds to the volume enhancing after injection of contrast product, possibly merged with a recent liver MRI.

The clinical target volume (CTV) is obtained by 3D expansion of 1 cm of the GTV (the expansion does not exceed the volume of the liver).

The planned target volume (PTV), including internal movements and repositioning uncertainties, is obtained by adding a margin of 1 cm laterally, anteroposteriorly and 1 cm head-to-toe in the event of treatment with respiratory or 1.5 to 2 cm in case of treatment in free breathing.

The OARs (see paragraph 4) included in the irradiation field must be drawn in their entirety for the calculation of the dose-volume histograms.

Irradiation technique

The total dose delivered to the isocenter is 54 Gy, at the rate of 3 Gy per fraction, 5 times a week for 3 to 4 weeks, for a total of approximately 18 sessions. The total dose (51 to 54 Gy) will be determined by the radiotherapist who will take care of the patient, according to the hepatic histograms-dose-volume constraints.

We recommend a multibeam conformal technique with a multileaf collimator or an arc therapy technique. Photon energy must be ≥ 6 MV.

Dose-volume histograms

Target volume

PTV: 95% of the volume covered at least by the isodose 95% (of the prescribed dose)

Note: histogram-dose-volume constraints are calculated from data published in the literature using a dose equivalent of 3 Gy / fraction, 5 sessions / week with an alpha / beta ratio of 2 for late effects.

Liver

total liver : dose moyenne ≤ 22 Gy

V 16 Gy ≤ 66%; V32 Gy ≤ 33%

Kidney

Rein droit + gauche : V 16 Gy ≤ 30%  ou sur rein unique V 16 Gy < 20%

Spinal cord

Dose max (1 cc) ≤ 42 Gy

Estomac- duodenum

V 25 Gy ≤ 25%, V 16 Gy ≤ 50 %

Small intestine, colon

Ces OAR n’étant pas dessinés en totalité, les histogrammes-dose-volume seront calculés en centimètre-cubes.

V 25 Gy ≤ 200 cc, V 16 Gy ≤ 400 cc

2.4. Summary of the results of non-clinical trials and clinical trials relevant to the biomedical research concerned

CRT appears to be an option for the treatment of HCC. A recent review summarizes its place [20].

Its efficacy, like the risk of RILD (Radiation Induced Liver Disease or radiation-induced hepatitis), depends on the dose delivered. In the old experience of the University of Michigan, 4% of patients had this complication and superior survival was observed in the event of irradiation greater than 75 Gy [21].

In France, a prospective non-randomized study by the group of F. Mornex and P. Merle involving 27 patients with a single HCC of less than 6 cm or two nodules of 3 cm demonstrated a complete response in 20 patients with a follow-up of 29 months. [22]. In a more recent study by the same group, the combination of classic TACE and RTC made it possible to obtain a complete response rate of 100% for HCCs < 5 cm, 60% for HCCs of 5-7 cm and 50 % for HCC > 7 cm. Very interestingly, local recurrences are rare (< 5%) [Philippe Merle, personal communication and abstract AASLD 2009].

Apart from the American and French experiences, the other studies are of Asian origin. In China, 203 patients with unresectable HCC, Child A or B were treated with TACE alone (n:=149), TACE then RTC (30-60 Gy divided by 2 Gy, n=54). The response rate and survival were better in the TACE RTC group in this non-randomized study [23]. Similar results are reported with less fractionated radiotherapy (48–60 Gy in 4–7.5 Gy per fraction). Other non-randomised trials including patients with large tumors and/or Child B cirrhosis also suggest increased activity of the TACE RTC combination [24] [25,26] [27] [28].

Finally, it appears that synchronization with the respiratory cycle decreases the volume of irradiated non-tumorous liver and consequently the occurrence of RILD [27,29].

## 2.5. Benefits and risks for patients

### 2.5.1. Benefits

#### Individual benefits

The combination of a TACE followed by a RTC should be superior to the reference treatment by a series of 2 or 3 TACE on the time without tumor progression or even on the survival of the patient.

Collective benefits

This trial, if its main objective is confirmed, should improve the treatment of HCC and reduce its cost. Indeed, each TACE requires 3 to 7 days of hospitalization, biological monitoring, anesthesia consultations and its effectiveness is all in all limited. It requires for most teams to be repeated. Radiotherapy is ambulatory and painless and its human and financial cost possibly lower than 2 sessions of TACE. This last point will be the subject of a medico-economic evaluation..

### 2.5.2. Risks for the patients

#### DC Beads TACE

TACE by DC Beads carries the known risks of classic lipiodol TACE: pain, asthenia, hepatic decompensation, hyperthermia, hepatic abscess, systemic effects of doxorubicin, accidental embolization of non-hepatic arteries (gastric, vesicular), complications of arterial catheterization, irradiation under fluoroscopy. These effects are currently well prevented and seem particularly infrequent when TACE is hyper-selective (and not total) and uses DC Beads.

Conformal external radiotherapy

CRT is a painless treatment. However, it can induce immediate or delayed side effects depending on the dose delivered (total and per session), the organ(s) irradiated, the volume irradiated and the "intrinsic radiosensitivity" of the patient. If necessary, the treatments for side effects are transient and purely symptomatic. The possibility of an RILD is all the more frequent when the hepatic function is degraded and when the dose administered to the non-tumorous liver is high. RTC could also irradiate the non-tumorous liver, increasing the possibility of induction of new HCCs. Nothing indicates this theoretical risk in the literature. For this reason it is the tumor progression on the whole of the liver and not the simple control of the HCC treated by the TACE DC Beads which is the primary criterion.

Psychological risks and constraints

The TACE DC Beads requires local anesthesia and several days of hospitalization. Most complications are transient and mild. Decompensation of liver function is the primary concern. It is prevented by selecting patients with good liver function.

RTC requires about 18 sessions spread over a period of 3-4 weeks. The trial induces the need to go frequently to the radiotherapy center and induces obvious constraints such as not working during this period. It also induces the need for repeated liver tests during the period of irradiation. Other examinations are those scheduled and carried out normally.

Research constraints

Patients in the control group have the usual treatment, namely 2 or 3 brief hospitalizations of 3 to 7 days, approximately every 8 weeks, for the performance of TACE by DC Beads without increased monitoring caused by the trial. The follow-up proposed thereafter is unchanged.

Patients in the RTC group will have a single hospitalization for the TACE DC Bead. In the 4 weeks following TACE, they will have to undergo approximately 18 outpatient radiotherapy sessions requiring as much transport.

Throughout the study, the patient will have to perform 9 blood tests as well as 8 scans. Patients in the control group will have to perform 3 electrocardiograms, 2 for patients in the RTC group.

During the study, he will have to answer 2 EORTC-QLQ C30 questionnaires (see appendix 9) before and after the treatment period.

Assessment of acute toxicity

Acute toxicity is assessed using NCI CTC AE criteria version 3.0 (Common Terminology Criteria for Adverse Events) (see appendix 12).

RTOG/EORTC Late radiotherapy morbidity score

This score can be used for toxicities appearing more than 90 days after the end of irradiation (see appendix 11).

# 3. Research objectives and judgment criteria

## 3.1. Research objective

## Primary criteria

Obtain hepatic tumor progression-free survival in group 1 (TACE DC Beads followed by CRT) 20% higher than in the control group 2 ( 2 TACE DC Beads (reference treatment) with 8 weeks delay) on tumor progression-free survival hepatic at 18 months.

Thus, we have 2 distinct treatment arms:

- - group 1: trial treatment: 1 session of TACE DC Beads associated with approximately 18 sessions of RTC

### - group 2: reference treatment: 2 or 3 sessions of TACE DC Beads

### 3.1.2. secondary criteria

- acute toxicity (within 90 days of treatment)

- late toxicity (after 90 days of treatment)

- quality of life until disease progression or until death

- local control rate

- overall survival

- the economic superiority of the TACE-RTC combination compared to the reference treatment

## 3.2. Statement of primary endpoint and secondary endpoints

### 3.2.1. Primary endpoint

The primary endpoint is the tumor progression time defined radiologically on the EASL (European Association for the Study of the Liver) criteria. Patients who died without progression are censored. This criterion (time to progression) is preferred to progression-free survival in the recommendations of JM Llovet. [9]

#### 3.2.1.1. Tumor Response

Before 1994, the evaluation of the anti-tumor effect of a treatment was generally determined in accordance with the WHO criteria established at the end of the 1970s. The WHO criteria evaluate variations in the size of tumor (using two-dimensional measurements) measurable and evaluable lesions. Based on the experience and knowledge gained, the WHO criteria were re-evaluated in the 1990s leading to the development of a new set of criteria described in Criteria for Assessing Response in Tumors solids (RECIST) [30]. RECIST assesses tumor size change using the sum of one-dimensional measurements of the largest diameters considering up to 5 target lesions per organ and accounting for non-measurable lesions. RECIST is currently accepted as a basis for evaluating anti-tumor activity in all types of solid tumors and is approved by regulatory authorities.

However, assessment of tumor response in HCC using the WHO or RECIST criteria does not take into account the extent of tumor necrosis that occurs after treatment. Extensive tumor necrosis cannot be paralleled with reduction in tumor diameter. Therefore, in 2000, the European Association for the Study of the Liver (EASL) recommended a modification of the WHO criteria for use in HCC [2]. The EASL consensus conference proposed that a viable tumor reduction was more appropriate. The use of EASL criteria is now accepted in the evaluation of treatment response in HCC[9] .

3.2.1.2. EASL Response Criteria

#### In accordance with the EASL criteria, responses are defined as follows:

#### Complete response (CR): complete disappearance of all viable tumors and no new lesions

#### Partial Response (PR): 50% decrease in viable tumor volume of all measurable lesions

#### Stable disease (ST): Neither CR nor PR

#### Progression (PG): 25% increase in size of one or more measurable lesions or appearance of new lesions

#### Objective response: RC and RP

#### 3.2.1.3. RECIST Response Criteria

#### In accordance with the RECIST criteria, responses are defined as follows:

#### Complete response (CR): disappearance of all target lesions (5 lesions) confirmed at 4 weeks

#### Partial Response (PR): 30% decrease from baseline

#### Stable disease (ST): Neither CR nor PR

#### Progression (PG): 20% increase in the smallest sum of measurements since the start of treatment or appearance of new lesions

#### Objective response: RC and RP

#### 3.2.1.4. Treatment Response Measures

Computed tomography (CT) and magnetic resonance imaging (MRI) are the best available and reproducible methods for measuring target lesions in patients with HCC.

The evaluation of the response to treatment of the irradiated site will be carried out according to the EASL criteria on thoracic or hepatic computed tomography (CT) by a review committee drawn from the investigators.

CT scans will be performed every 12 weeks after the first chemoembolization. The response criteria are defined on the change in diameter of the total viable tumor volume:

Complete response (CR): complete disappearance of all viable tumors and no new lesions

Partial Response (PR): 50% decrease in viable tumor volume of all measurable lesions

Stable disease (ST): Neither CR nor PR

Progression (PG): 25% increase in size of one or more measurable lesions or appearance of new lesions

In the context of this trial, the objective response includes the rate of complete responses and partial responses. At the end of the evaluation of the response, the patient will be classified in one of the following 8 categories:

1 - complete response (CR)

2 - partial response (RP)

3 - stable disease (ST)

4 - progress (PG)

5 - early neoplastic death (DCD K)

6 - early death from toxic causes (DCD Tox)

7 - early death from other cause (DCD X)

8 - unknown (not evaluable, insufficient data) (I)

All patients who met the eligibility criteria should be included in the primary analysis of response rates.

Groups 4.5 and 8 should be considered treatment failure.

#### 3.2.1.5. Vérification de la réponse tumorale

Assessments of tumor response (including size and percentage of necrosis) and presence of new lesions will be recorded in the e-CRF by the investigators. Copies of the scans in the form of CDs will be submitted to the investigators of the various centers for proofreading, without knowledge of the treatment arm of the patients or the opinion of the investigator of the center concerned, according to a circuit which will be defined later.

### 3.2.2. Secondary endpoints

# - Overall survival

# - Rate of CR, PR and ST at 6, 12 and 18 months of follow-up

# - Side effects

# - EORTC QLQ C30 quality of life questionnaire

# - Medico-economic study4. Design of research

# 4.1. General research methodology

# 4.1.1. Experimental plan

This is a multicenter, controlled, open, randomized trial in two balanced 1:1 parallel groups, seeking to demonstrate in intention to treat, that the association of a TCR after a course of hepatic TACE by DC Beads is superior for tumor progression time to the usual treatment with 2 to 3 courses of TACE using DC Beads.

# 4.1.2. Course of the study

In each center, patients with an indication for TACE DC Beads for the treatment of HCC will be identified for the trial criteria in a multidisciplinary consultation meeting or radiology staff.

Patients for whom an indication for TACE DC Beads is made will have their imaging examined by radiotherapists in order to judge the feasibility of RTC. If so, patients will be presented with the principle of the trial. The scanners will be kept by the investigators (sheet or digital formats) for the study to assess the endpoints.

# 4.1.3. Etude médico-économique

Responsable de l’étude médico-économique :

Pr Isabelle DURAND-ZALESKI

Economie de la santé

URC Eco, AP-HP

Hôpital Henri Mondor

Créteil

Tél: 01 49 81 40 35

Fax: 01 49 81 36 74

isabelle.durand-zaleski@hmn.aphp.fr

4.1.3.1. Type of costs taken into account

The cost study will be limited from the point of view of the community, being limited to the estimation of direct medical and non-medical costs, excluding indirect costs.

1. Direct medical costs

• treatment phase: irradiation (preparation, sessions, quality controls), day hospitalization

• hospital follow-up phase: possible hospitalization for complications up to 1 month following the treatment

2. Direct non-medical costs

Direct non-medical costs are represented by transport to get to the place of treatment.

These costs will be evaluated retrospectively, from the start of radiotherapy and over a period of one month following the end of treatment..

4.1.3.2. Cost calculation specifications

The cost estimate will mobilize the micro-costing method, a method which is based on detailed observation for each patient of all the resources consumed by the patient.

The data collected will follow the different stages of radiotherapy. For each patient, the following phases will be distinguished and documented:

**RTC and TACE:**préparation du traitement (système de contention, acquisition d’images TDM, planification du traitement et transfert des données vers l’accélérateur)

séances d’irradiation

• machine quality control

• patient quality control

• Patient transport

The main cost elements that will be taken into account for each of these phases are as follows:

• medical and paramedical staff costs

• equipment depreciation costs

• equipment maintenance costs

• costs of consumables used

The consumption of resources linked to each of these phases will be identified and quantified for each patient, at the same time as the clinical data, based on the economic questions included in the observation book and documented by the clinical research associates (CRA):

• time of each category of medical and non-medical personnel

• consumables used

• type of equipment and duration of immobilization

The unit costs used to value this data will be obtained from a specific questionnaire completed by each center:

• specification of the type of equipment used with purchase price, lifespan, number of actual annual hours of use

• annual cost of maintenance

• average annual number of treatment starts with the equipment regardless of the irradiation technique.

Follow-up phase:

All hospitalizations for treatment-related complications will be listed by the CRA in the observation book:

• reason and duration of hospitalization

• procedures performed during hospitalization

This follow-up will take place up to 1 month after the end of the treatment.

Transport:

Clinical Trial Technicians (TEC) will be responsible for interviewing patients regarding:

- the type of vehicle used

- the number of round trips between the home and the place of treatment.

- The monetary valuation will be carried out on the basis of the current social security reimbursement rate.

## Schéma général de l’étude

# 5. Study population

## 5.1. Description of the population to be studied

### 5.1.1. Description of population

Patients must be HCC carriers who cannot benefit from resection, RFA or an immediate indication for TH, have an indication for TACE treatment by DC Beads and can technically undergo HCC irradiation.

### 5.1.2. Description and number of subjects planned for the sample

It is planned to include 174 patients over 24 months. The calculation of the number of patients is detailed in § 8.2.2

Before the inclusion of the patient in the trial, the investigator will explain the study to him in a complete way. An information note (see appendix 4) giving all the details of the study will be provided to the patient for reading at least 24 hours before the start of the study and the patient will keep a copy. After reading this information and having been encouraged to ask questions, the investigator will ask him to give his consent in writing (see appendix 5) by writing the date and his name in his own hand. The investigator will countersign this form which he will date with his name and will give the patient a copy of the consent signed by both parties.

Consent forms bearing the patient's name will need to be checked on site. The original will be kept by the investigator in the study file.

There will be no inclusion of patients who will not be informed of the diagnosis of their disease.

### 5.1.3. Recruitment methods

Patients will be recruited by the 9 participating hepato-gastroenterology departments, all involved in HCC screening and treatment. These centers ensure the monitoring of thousands of cirrhotic subjects and are increasingly detecting (progress in imaging) HCC at a stage allowing curative or palliative therapy to be envisaged.

List of clinical investigators in charge of patient recruitment in appendix

## 5.2. Inclusion criteria

Age ≥ 18 years old

ECOG 0-1

Life expectancy ≥ 6 months

HCC proven histologically or according to radiological and biochemical criteria (EASL/AASLD) in cirrhotic patients

Maximum lesion ≤ 9 cm

Not eligible for surgery, percutaneous therapy

Child-Pugh A or early B (7 points for the Child-Pugh score)

AST and ALT < 7 x ULN

Technical possibility of conformal irradiation of the tumor mass

Technical possibility of treatment with DC Beads

The entire tumor mass must be able to receive the DC Beads

Written informed consent signed by the patient

Attention: the inclusion criteria must be checked before each TACE.

## 5.3. Non-inclusion criteria

- Metastatic HCC disease

Uncontrolled viral B replication

Possibility of liver transplantation within 12 months

History of abdominal radiotherapy (including lipiciocis or yttrium90)

Lack of means or refusal to use effective means of contraception for men or women of childbearing age

Pregnancy and lactation

Contraindication to DC Beads chemoembolization or conformal external radiotherapy

Any other concomitant experimental treatment

Contraindication of doxorubicin

# 6. Treatments used during the study

## 6.1. Description of the treatment(s) necessary to carry out the research

### 6.1.1. For TACE

The treatments used will be microspheres called DC Beads loaded with 37.5 mg/ml of doxorubicin for a total dose of 150 mg.

During hepatic arteriography, 2 or 4 ml of DC Beads (1 to 2 vials of microspheres with a diameter of 100-300 µm or possibly 1 to 2 vials of microspheres with a diameter of 300-500 µm in the case of a shunt) will be injected into the patient.

See the EuroPharmat information file in appendix 13.

### 6.1.2. For the RTC

## 6.2. Authorized treatments

All treatments for extra-hepatic conditions will be continued.

Therapies treating the underlying non-tumor liver disease will be continued.

## 6.3. Prohibited treatments

Sorafenib or any other chemotherapy will be stopped on definitive inclusion. They will be resumed in the event of tumor progression. This point could be amended later.

Treatments including interferon alfa are not authorized for the duration of the irradiation.

## 6.4. Treatment adherence monitoring methods

The bottles of DC Beads used will be kept by the pharmacy of the investigating establishment for accounting during monitoring visits.

The destruction of the bottles will only be authorized after written agreement from the promoter.

A certificate of destruction must be provided in return.

## 6.5. Storage conditions for investigational drugs

### 6.5.1. Description of storage at the pharmacy

DC Beads should be stored in a cool, dry, dark place in their original packaging. They should not be frozen.

The validity period of the product is 4 years.

The product does not require the presence of temperature indicators.

The particles will be loaded at the pharmacy before use by the radiology department.

They can be stored loaded for 14 days in the refrigerator (2 to 8°C).

Contact details of the responsible pharmacy:

Hotel-Dieu Pharmacy – u.p.c.o

8th floor south wing

1 place Alexis Ricordeau

44093 Nantes Cedex 1

Tel: 02 40 08 46 37

### 6.5.2. Description of storage in the service

The charged particles mixed with the contrast product can be stored for 7 days in the refrigerator (2 to 8°C).

# 7 Conduct of the study

Study and analysis techniques

## 7.1.1. Description of the measures taken to reduce and avoid bias, including in particular

### 7.1.1.1. The draw

Patients are assigned to a treatment group by central Internet randomization (minimization method) stratified by center on:

- the existence of treatment with sorafenib at the time of inclusion (yes or no),

- the existence of previous percutaneous or arterial treatment (yes or no),

- the uni-nodular character (yes or no).

The investigator is not involved in the choice of treatment group.

Randomisation will be carried out at the time of the inclusion visit, i.e. within 4 weeks prior to the start of therapy. This in order to be able to program the radiotherapy sessions in the experimental group.

According to the minimization procedure, the patient will be randomized in such a way as to balance the minimization factors. The objective is for the two treatment groups to be correctly balanced for all of the minimization factors.

### 7.1.1.2. Blinding methods

Not applicable

### 7.1.1.3. Arrangements implemented to maintain blinding and procedures for unblinding, if applicable

The rereading of the imaging will be carried out by the rereading committee without the knowledge of the arm on all the scanners.

Each referring radiologist in the centers will be part of the scanner review committee. A circuit of scanners between the centers will be organized so that each center carries out the proofreading of the scanners of only one other center.

# Study schedule

## Inclusion visit and Randomisation from week –4 to week 0

Inclusion visit

The investigating doctor will have to carry out various checks and collect several pieces of information:

- Information to the patient and signature of the consent

- Verification of eligibility criteria

- Fill in the demographic data

- List the concomitant pathologies

- List associated processing

- Inform the history of the disease

- List previous treatments for HCC

- Assess the patient's Child-Pugh score

The investigating physician will carry out a clinical examination of the patient with the ECOG score (see appendix 10).

The patient must, at this visit, have a biological sample for an evaluation of his liver function (ALAT, ASAT, total and conjugated bilirubin, factor V, INR, albuminemia, alpha-foeto protein) and his blood count (NFS ) and platelets less than 4 weeks old. It will be the same for the thoraco-abdominal scanner.

During this visit, the doctor will perform an electrocardiogram (ECG) to assess his heart function.

Any woman of childbearing age should take a urine pregnancy test.

During this visit, patients must answer the EORTC QLQ-C30 quality of life assessment questionnaire (see appendix 9).

Following this visit, the doctor will have to plan with his patient his hospitalization for the 1st course of TACE within 4 weeks following inclusion. If possible, he can also schedule subsequent TACE or RTC sessions.

## Week 0

This visit is common for the 2 groups. It corresponds to the hospitalization of the patient for 3 to 7 days to make the 1st TACE.

During this visit, the doctor checks the patient's eligibility criteria, reviews the concomitant pathologies, adverse events and associated treatments. He performs a clinical examination of the patient with the ECOG score and evaluates the Child-Pugh score.

A biological sample is taken from this patient (ALAT, ASAT, total and conjugated bilirubin, factor V, INR, albuminemia, NFS-platelets) before carrying out the TACE.

Every woman of childbearing age takes a urine pregnancy test.*Groupe 1*

Patients in group 1 have a course of approximately 18 radiotherapy sessions spread over 3 to 4 weeks.

Regularly, patients have visits spread over 4 months to assess different parameters:

## Week 2:

The doctor reviews the concomitant pathologies, adverse events and associated treatments. He performs a clinical examination of the patient with the ECOG score and an ECG.

The physician assesses acute toxicity using the CTC-NCI scale.

Any woman of childbearing age should take a urine pregnancy test.

## Week 4:

The doctor reviews the concomitant pathologies, adverse events and associated treatments. He performs a clinical examination of the patient with the ECOG score. It evaluates the Child-Pugh score.

A biological sample is taken from this patient (ALAT, ASAT, total and conjugated bilirubin, factor V, INR, albuminemia, NFS-platelets) before his radiotherapy session.

The patient must perform a thoraco-abdominal CT scan.

The physician evaluates the response according to the EASL criterion and the acute toxicity according to the CTC-NCI scale.

Any woman of childbearing age should take a urine pregnancy test.

## Week 6 and 8:

The doctor reviews the concomitant pathologies, adverse events and associated treatments. He performs a clinical examination of the patient with the ECOG score.

The physician assesses acute toxicity using the CTC-NCI scale.

Any woman of childbearing age should take a urine pregnancy test.

## Week 12:

The doctor reviews the concomitant pathologies, adverse events and associated treatments. He performs a clinical examination of the patient with the ECOG score. It evaluates the Child-Pugh score.

A biological sample is taken from this patient (ALAT, ASAT, total and conjugated bilirubin, factor V, INR, albuminemia, alpha-feto-protein, NFS-platelets) before his radiotherapy session.

The patient must perform a thoraco-abdominal CT scan.

The physician evaluates the response according to the EASL criterion and the acute toxicity according to the CTC-NCI scale.

Any woman of childbearing age should take a urine pregnancy test.

## Week 16:

The doctor reviews the concomitant pathologies, adverse events and associated treatments. He performs a clinical examination of the patient with the ECOG score.

The doctor assesses late toxicity according to the RTOG scale.

Any woman of childbearing age should take a urine pregnancy test.

# Group 2

Patients in group 2 have 2 or 3 chemoembolizations, including 1 in common with group 1.

Regularly, patients have visits spread over 4 months to assess different parameters:

## Week 4:

The doctor reviews the concomitant pathologies, adverse events and associated treatments. He performs a clinical examination of the patient with the ECOG score. It evaluates the Child-Pugh score.

A biological sample is taken from this patient (ALT, AST, total and conjugated bilirubin, factor V, INR, albuminemia, alpha-feto-protein, NFS-platelets).

The patient must perform a thoraco-abdominal CT scan.

The physician evaluates the response according to the EASL criterion and the acute toxicity according to the CTC-NCI scale.

Any woman of childbearing age should take a urine pregnancy test.

## Week 8:

Patients are hospitalized for 3-7 days to perform their 2nd TACE. This 2nd cure can only be carried out after restoration of liver function.

During this hospitalization, the doctor reviews the concomitant pathologies, adverse events and associated treatments. He performs a clinical examination of the patient with the ECOG score and an ECG.

The physician assesses acute toxicity using the CTC-NCI scale.

Any woman of childbearing age should take a urine pregnancy test.

## Week 12:

The doctor reviews the concomitant pathologies, adverse events and associated treatments. He performs a clinical examination of the patient with the ECOG score. It evaluates the Child-Pugh score.

A biological sample is taken from this patient (ALT, AST, total and conjugated bilirubin, factor V, INR, albuminemia, alpha-feto-protein, NFS-platelets).

The patient must perform a thoraco-abdominal CT scan.

The physician assesses acute toxicity using the CTC-NCI scale.

Any woman of childbearing age should take a urine pregnancy test.

Week 16:

Patients are hospitalized for 3-7 days to perform their 3rd TACE if required. This 3rd cure can only be carried out after restoration of liver function.

The doctor reviews the concomitant pathologies, adverse events and associated treatments. He performs a clinical examination of the patient with the ECOG score and an ECG.

The physician evaluates the response according to the EASL criterion and the late toxicity according to the RTOG scale.

Any woman of childbearing age should take a urine pregnancy test.

Followed

The follow-up of the patients is the same in the 2 groups.

## Week 24:

The doctor reviews the concomitant pathologies, adverse events and associated treatments. He performs a clinical examination of the patient with the ECOG score. It evaluates the Child-Pugh score.

A biological sample is taken from this patient (ALT, AST, total and conjugated bilirubin, factor V, INR, albuminemia, alpha-feto-protein, NFS-platelets).

The patient must perform a thoraco-abdominal CT scan.

The physician evaluates the response according to the EASL criterion and the late toxicity according to the RTOG scale.

During this visit, patients will have to answer the EORTC QLQ-C30 quality of life evaluation questionnaire.

Follow-up every 3 months for 1 year (4 visits):

The doctor reviews the concomitant pathologies, adverse events and associated treatments. He performs a clinical examination of the patient with the ECOG score. It evaluates the Child-Pugh score.

A biological sample is taken from this patient (ALT, AST, total and conjugated bilirubin, factor V, INR, albuminemia, alpha-feto-protein, NFS-platelets).

The patient must perform a thoraco-abdominal CT scan.

The physician evaluates the response according to the EASL criterion and the late toxicity according to the RTOG scale..

|  | Inclusion  Et  randomisation | Traitement | | | | | | | | | | | Suivi | |
| --- | --- | --- | --- | --- | --- | --- | --- | --- | --- | --- | --- | --- | --- | --- |
|  | S-4 à S0 | Groupe 1 et 2  TACE^d^ | Groupe 1 : 18 séances de radiothérapie sur 3-4 semaines | | | | | | Groupe 2 : TACE^d^ | | | | S24 | Suivi tous les 3 mois pendant 1 an |
|  |  | S0^b^ | S2 | S4 | S6 | S8 | S12 | S16 | S4 | S8^c^  TACE | S12 | S16 ^c^  TACE |  |  |
| Information patient et signature consentement | X |  |  |  |  |  |  |  |  |  |  |  |  |  |
| Vérification des critères d’éligibilité | X | X |  |  |  |  |  |  |  | X |  | X |  |  |
| Données démographiques | X |  |  |  |  |  |  |  |  |  |  |  |  |  |
| Pathologies concomitantes | X | X | X | X | X | X | X | X | X | X | X | X | X | X |
| Evènements indésirables |  | X | X | X | X | X | X | X | X | X | X | X | X | X |
| Traitements associés | X | X | X | X | X | X | X | X | X | X | X | X | X | X |
| Examen clinique avec score ECOG | X | X | X | X | X | X | X | X | X | X | X | X | X | X |
| Histoire de la maladie | X |  |  |  |  |  |  |  |  |  |  |  |  |  |
| Traitements antérieurs pour le CHC | X |  |  |  |  |  |  |  |  |  |  |  |  |  |
| Score de Child-Pugh | X | X |  | X |  |  | X |  | X |  | X |  | X | X |
| NFS-plaquettes | X^a^ | X |  | X |  |  | X |  | X |  | X |  | X | X |
| ALAT ASAT bilirubine totale et conjuguée facteur V INR albuminémie | X^a^ | X |  | X |  |  | X |  | X |  | X |  | X | X |
| Alpha foeto protéine | X^a^ |  |  |  |  |  | X |  |  |  | X |  | X | X |
| Β-HCG urinaire | X | X | X | X | X | X | X | X | X | X | X | X |  |  |
| ECG | X |  | X |  |  |  |  |  |  | X |  | X |  |  |
| TDM thoraco-abdominal | X^a^ |  |  | X |  |  | X |  | X |  | X |  | X | X |
| Evaluation de la réponse selon critères EASL |  |  |  | X |  |  | X |  | X |  | X |  | X | X |
| Evaluation qualité de vie  EORTC QLQ-C30 | X |  |  |  |  |  |  |  |  |  |  |  | X |  |
| Evaluation toxicité aiguë selon l’échelle CTC-NCI |  |  | X | X | X | X | X |  | X | X | X |  |  |  |
| Evaluation toxicité tardive selon l’échelle RTOG |  |  |  |  |  |  |  | X |  |  |  | X | X | X |
| Etude médico-économique | X | X | X | X | X | X | X | X | X | X | X | X | X | X |

# Flow-chart de l’étude TACERTE

a : TDM et biologie de moins de 4 semaines

b : le traitement doit débuter le plus tôt possible, sans dépasser 4 semaines après l’inclusion.

c : en fonction du temps jusqu’à restitution de la fonction hépatique

d : TACE : Chimioembolisation par DC Beads - pas d'anesthésie générale obligatoire ni recommandée.

## 7.3. Identification of all the data to be collected directly in the observation notebooks, which will be considered as source data

### All the data will be the subject of an observation book or Case Report Form (CRF). (see appendix 14 and 15)

## Rules for stopping a person's participation

7.4.1 Criteria for premature discontinuation of treatment or exclusion of a person from research

If a patient permanently discontinues treatment, the reasons for this discontinuation will be reported in the CRF.

These reasons can be:

- Decision of the investigator or sponsor

- Withdrawal of consent or refusal of treatment by the patient

- Occurrence of grade 4 toxicity during treatment, requiring discontinuation of treatment

- Tumor progression

- Inability of the patient to continue the study (inability to respect the constraints of respiratory servoing (for centers that use it), intercurrent illness, significant deterioration in general condition)

- Loss of sight of the patient

-Death

Insofar as the patient does not definitively stop the study, he cannot participate in any other therapeutic trial related to his illness.

### Modalités d’arrêt prématuré du traitement ou d’exclusion d’une personne de la recherche

### Withdrawals from studies can only be effective after confirmation by the investigator and the sponsor. These study exits are always final. An evaluation report corresponding to the half-yearly report will be carried out at the time of departure.

### When a patient leaves the study, subsequent data concerning him will no longer be collected (except for serious adverse events).

### In the event of loco-regional progression or new metastatic localization, the patient may be treated according to the standard treatment methods for metastatic colorectal cancer.

### 7.4.3. Search stopping criteria

The date of the end of the research will be the date of the end of the follow-up (48 weeks after the end of the treatment) of the last patient followed in the study. However, the research may be interrupted early (Afssaps decision, monitoring committee, ineffectiveness, toxicity, etc.).

# 8. Data management and statistics

## 8.1. Collection and processing of study data

### 8.1.1. Data collection

### One CRF will be created per patient. All information required by the protocol must be provided in the CRF. He will take up the different stages of patient care in the protocol. It must include the data necessary to confirm adherence to the protocol and identify major deviations from the protocol.

### The paper observation book

### Depending on the centre, the investigator or a CRA will keep the CRFs up to date.

### The data must be copied clearly and legibly.

### Missing data will be notified by the inscription DM (missing data).

### Erroneous data will be clearly crossed out and new data copied alongside with the date and the corrector's initials.

### The anonymity of the subjects will be ensured according to the rules defined in 10.4.3 All personal data will be erased.

### The notebooks will be dated and signed when the patient leaves the trial in order to validate the data.

### The medico-economic study will be the subject of a paper CRF alone unlike the rest of the study which will have an electronic CRF.

### The electronic observation book

### The version of electronic CRF will be produced by the data-management of the Nantes University Hospital research office.

### Data collection will be carried out directly by the investigator or the TEC of the clinical team of each participating investigation center, using an electronic CRF (eCRF) accessible from the website https://www.hugo-online.org and developed by the Nantes University Hospital Promotion Unit with the Capture System software from Clinsight (installed pack version: 5.05.4102).

### Each person (investigator, TEC, Project Manager, CRA) will have a personal user account related to their role (profile) assigned to them by the trial sponsor. The creation and administration of computer accounts will be provided by the Data Manager in charge of the trial.

### Each user must change their initial password on the first connection: encrypted password of at least 8 alphanumeric characters, password validity period: 30 days, standby after 15 minutes of inactivity on the application, blocking of the user account after 3 failed logins.

### An entry and navigation guide will be provided to the investigator and the TEC in charge of data collection. The Data Manager in charge of the trial will provide telephone assistance in the event of a problem related to the use of the Capture System application or the eCRF

### The anonymity of the subjects will be ensured according to the rules defined in 10.4.3 All personal data will be erased.

### 8.1.2. Data processing

### The collection of clinical data will be based on the establishment of a clinical database and the creation of input masks like the observation book in accordance with the protocol and regulations currently in force.

### The structure of the database and entry screens will be approved by the trial sponsor.

### 8.1.3. Data Archiving

All study notebooks and documents must be kept in a locked cabinet for 15 years. This information must be kept by the sponsor and the investigator.

## Statistiques

Statistical Analysis Manager

Loic Campion, MD

Biostatistician

e-mail: l-campion@nantes.fnclcc.fr

• Biostatistics and Integrated Biology Unit

René Gauducheau Center - Center for the Fight Against Cancer Nantes

Boulevard Jacques Monod - 44805 Saint-Herblain Cedex

Tel: 02.40.67.99.00 - www.centregauducheau.fr

• Cancer Research Center - Inserm U892 (Team 11)

Institute of Biology - 9, quai Montcousu - 44093 Nantes Cedex 018.2.1. Description des méthodes

#### Covariates

The variables studied will be:

• demographics: age, sex, weight, treatment center

• initial descriptions:

o etiology of the underlying liver disease

o therapeutic modalities already implemented

o clinical, paraclinical or biological related to the description of the disease: tumor mass (number of nodules, maximum size, sum of diameters, portal tumor thrombosis, etc.),…

o current treatment modalities

• follow-up descriptions: clinical, paraclinical or biological related:

o efficiency

o the side effects of the treatment

o and the progression of the disease (relapse, etc.)

• medico-economic

• quality of life

## Statistical tests used

Statistical analyzes will be descriptive and comparative, all done with stratification on potential confounding parameters: center, existence of prior percutaneous or arterial treatment (yes or no), treatment with sorafenib (Y/N) and unifocal nature (WE).

Comparisons of the various groups of interest will be made for numerical data by Student's t-test or ANOVA (or non-parametric equivalents of Mann-Whitney or Kruskal-Wallis if necessary) and for discrete data by the test of Pearson's Chi² (or Fisher's exact test if necessary).

Survival curves will be plotted using the Kaplan-Meier method and comparisons of survival curves will use the logrank test. Multi-parametric analyzes for the search for independent prognostic factors for progression-free survival will be carried out using the Cox regression model with stratification.

The analyzes will be done by intention to treat and per protocol following a bilateral scheme. The p of significance limit will be set at 5%.

Analysis software will be SAS 9.2 (SAS Institute Inc., Cary, NC, USA) and Stata SE 10.

### Analyses intermédiaires prévues

Due to the ethical importance of rapidly detecting either a clearly superior efficacy of the experimental group, or a clearly insufficient tolerance of the experimental arm, an interim analysis will be carried out as soon as half of the patients included are evaluable (1 year follow-up). The Peto-Haybittle method, using a very low alpha threshold for the intermediate analysis so as not to conclude too hastily, will make it possible not to reduce the overall risk too much and therefore the final alpha threshold. Thus, if the statistical tests of the intermediate analysis are carried out at the threshold of 0.001%, the final threshold is then little different from 5%.

### 8.2.2. Statistical justification of the number of inclusions

The main judgment criterion is the progression time defined radiologically on the EASL criteria. Patients who died without progression are censored. This criterion (time to progression) is preferred to progression-free survival in the recommendations of JM Llovet[9].

The available data allow us to estimate that 35-40% of patients have no tumor progression at 18 months in the reference group (2-3 TACE DC Beads).

The hypothesis tested is, in intention to treat, the 20% increase in patients without tumor progression at 18 months, ie 55-60% in the experimental arm with radiotherapy.

Given these elements, in a bilateral situation, with a type I risk α of 5%, a power of 80%, an inclusion period of 24 months and a minimum follow-up period of 18 months, the number of patients required is N=75 per group.

To take account of those lost to follow-up (estimated 15%), it will be necessary to include 87 patients per group, ie 174 in total.

### 8.2.3. Expected level of statistical significance

The various tests will be carried out in a bilateral situation with a limit significance p set at 5%.

### 8.2.4. Statistical search stop criteria

Tumor progression-free survival (TTP), overall survival and toxicity will be analyzed during the interim analysis. In the event of a highly significant difference (p<0.001) between the 2 groups during the interim analysis, the independent monitoring committee decides whether or not to continue the study.

### 8.2.5. How to account for missing, unused, or invalid data

Particular attention will be paid to minimize missing data as much as possible. All randomized patients must be followed up until their tumor progression (primary objective), their death or the end of the trial, regardless of their "compliance" with the treatment of their group to which they were assigned by randomization, in order to have the set of primary endpoints.

In the event of missing data which would nevertheless occur on the variables studied other than the primary endpoint, their treatment will depend on the frequency. If the frequency of missing items is less than 10-15%, the variable in question will be tested according to the usual maximum bias procedure. If the frequency of missing variables for the variable is greater than 10-15%, the variable will not be analyzed due to the excessive risk of bias.

### 8.2.6. Choice of people to include in the analyzes

All randomized subjects will be analyzed. The analyzes will be done by intention to treat and per protocol.

### Randomisation

A patient is included in the study when the patient meets the inclusion/exclusion criteria and consent is obtained. The investigator then connects, via the internet, to the TenAlea software which centralizes the inclusion/randomization of patients. https://fr.tenalea.net/chunantes/

The randomization list as well as the implementation of this centralized randomization system is under the responsibility of the Biostatistics department of the Nantes University Hospital. Each patient entering the trial will receive the treatment provided by the lottery. This draw will be balanced between the two treatment arms.

The inclusion number as well as the patient's treatment arm will be communicated by e-mail to the investigating center as well as to the research office.

# 9. Vigilance and management of adverse events

## 9.1. Definitions

### 9.1.1. Adverse events (EvI)

An adverse event is defined as any harmful manifestation in a patient or a participant in a clinical trial, and which is not necessarily related to the treatment planned in the clinical trial.

All adverse events encountered during the study, which are noted by the doctor or reported by the patient, will be recorded in the observation notebook in the section provided for this purpose.

The intensity of adverse events will be rated according to the criteria chosen when drafting the protocol *(include the chosen evaluation criteria in the appendix)* . For any event not rated in the chosen classification, the rating will be as follows:

1 = benign

2 = moderate

3 = severe

4 = life-threatening

### 9.1.2. Adverse Effects (AE)

An adverse event is considered to be suspected for any adverse event for which a causal link, whatever its importance (doubtful, plausible, possible, certain) can be considered either with the treatment under study or with the comparator or the protocol.

Relationship to the treatment under investigation

The relationship of an adverse event to the investigational product will be determined by the investigation based on its clinical judgment, using the following definitions:

Definite relationship:

Adverse event appearing at a reasonable interval of time after administration of a product under investigation, corresponding to a known effect of the product, improving following discontinuation of treatment, and reappearing following renewed exposure to the product (reintroduction). If, given the nature of the adverse event, reintroduction is impossible, only a probable relationship can be concluded (see below).

Likely relationship:

Adverse event appearing at a reasonable interval of time after administration of a product under investigation, corresponding to a known effect of the product under investigation, improving following its discontinuation, and which cannot be reasonably explained by known clinical conditions of the patient or by other therapies.

Unlikely relationship:

Adverse event occurring within a reasonable time interval after administration of a product under investigation, corresponding to a known effect of this product, but which could be due to the clinical condition of the patient or to other medicinal products.

Very unlikely relationship:

Adverse event appearing with no obvious temporal relationship to the administration of the product, and/or presence of a possible relationship with the clinical condition of the patient or with another concomitant treatment.

No relationship:

Adverse event for which there is sufficient information indicating that the etiology is not related to the product. Another etiology must then be established.

### 9.1.3. Serious adverse events or effects EVIG/SUE

An AE is considered an SAE when it:

* leads to death

* is life-threatening

* results in temporary or permanent incapacity or disability

* requires or prolongs patient hospitalization

* results in a congenital or neonatal abnormality

* is medically important (meaning: requires management to prevent aggravation to one of the above stages)

### 9.1.4. Expected adverse effects or events

An expected adverse event (AE A) is an event already mentioned in the most recent version of the investigator's brochure or in the most recent summary of product characteristics for medicinal products which already have a marketing authorization.

These expected serious adverse effects or events will be the subject of a deferred declaration by the sponsor to the competent authorities.

### 9.1.5. Unexpected serious side effects

An unexpected serious adverse event (SAE I) is an event whose nature, severity, frequency or course does not match the information in the most recent investigator's brochure or the most recent summary of product characteristics for medicinal products with marketing authorization.

These unexpected serious adverse effects will be the subject of a declaration within 7 or 15 days following their becoming known by the promoter to the competent authorities.

## 9.2. Security Assessment Parameters

Each patient will benefit from clinical follow-up, regular evaluation of side effects (according to standardized scales) and biological follow-up according to a pre-established schedule (see flow-chart page 31).

### Specific evaluation criteria related to security

Patients will have a particular evaluation of possible toxicities due to the treatment. There will be :

- acute toxicity assessments according to the CTC-NCI scale (see appendix 12),
- assessments of late toxicity according to the RTOG scale (see appendix 11),

according to a pre-established schedule.

### Planned methods and schedule for measuring, collecting and analyzing security assessment metrics

Any event relating to the study will be reported to the sponsor by the investigators. Similarly, quality control of files will be implemented by the promoter through monitoring visits.

These events will be detected during consultations and, if necessary, by telephone or email contact with local investigators.

## 9.3. List of expected ARs

### 9.3.1 Concerning radiotherapy

- Esophageal ulceration responsible for bleeding (less than 1% of cases)
- Severe spasmodic cough resistant to treatment
- Gastroduodenal ulceration responsible for bleeding (less than 2% of cases)
- Inflammatory colitis with more than 7 bowel movements per day
- Febrile syndrome > 40°C occurring after tumor necrosis, and requiring hospitalization for investigation

### About DC Beads Chemoembolization

EI A due to DC Beads:

- Unwanted reflux or passage of DC Beads into normal arteries adjacent to the target lesion or through the lesion to other arteries or arterial beds
- Untargeted embolization
- Pulmonary embolization
- Ischemia at an unwanted site
- Capillary bed saturation and tissue damage
- Ischemic stroke or ischemic infarction
- Rupture of a vessel or lesion and hemorrhage
- Neurological deficits including cranial nerve palsy
- Vasospasm
- Death
- Recanalization
- Foreign body reaction requiring medical intervention
- Infection requiring medical intervention
- Formation of a clot at the tip of the catheter and subsequent mobilization

**AR A due to doxorubicin :** Vidal Dictionary, 2010 edition (Summary of Product Characteristics Adriblastine ^®^ )

Note: It is necessary to specify that the doxorubicin used in DC Beads acts locally on the tumor and only very rarely exhibits the systemic effects found when doxorubicin is injected intravenously.

As with other DNA-damaging anticancer agents, myelodysplastic syndromes and acute myeloid leukemias have been observed after combination therapy including doxorubicin.

With topoisomerase II inhibitors, a higher than expected incidence of secondary leukaemias presenting as de novo leukaemias LAM2, LAM3, LAM4 has been reported. Such forms may have a short latency period (1 to 3 years). These forms, accessible to curative treatment, require early diagnosis and appropriate treatment with curative intent.

Adriblastine ^®^ can give rise to undesirable effects:

- stomatitis,
- bone marrow hypoplasia in about two thirds of patients,
- rapidly regressive immunosuppression,
- alopecia in 90% of cases but reversible when treatment is stopped,
- amenorrhea, azoospermia.

The onset of febrile attacks, nausea, vomiting, abdominal pain and diarrhea have also been reported. But these manifestations are transient and do not pose a serious therapeutic problem.

Some changes in the ECG may appear: arrhythmias, prolongation of the QT space in particular; acute arrhythmias may occur within hours of the injection. Frequent ECG checks, possibly supplemented by a 24-hour recording (Holter method) should make it possible to clarify the meaning. Any associated electrolyte disturbances (hypokalaemia, hyponatraemia) must be corrected. In some cases, severe heart failure, unresponsive to usual treatment, may occur. These reactions are rare in patients who have received a total dose of less than 550 mg/m ^2^ , they are more frequent above this dose and can in this case reach 27% of patients.

## 9.4. Management of adverse events

### 9.4.1. Notification of SAEs

Any EI that meets the definition of SAE requires the completion of a SAE declaration form **whether expected or not expected** . The investigator must check that the information provided on this sheet is precise and clear (do not use abbreviations).

The SAE must be reported immediately (within 24 hours of its detection by the investigator) to the sponsor by fax (Clinical Research Promotion Unit, Nantes University Hospital, Fax 02 40 08 71 67).

### 9.4.2. Declaration to competent authorities

After receiving notification of an SAE, the sponsor assesses whether the SAE is expected or unexpected. He undertakes to transmit to the authorities (EMEA, AFSSAPS, etc.) SAEs I within 7 days for deaths and SAEs involving a life-threatening threat (with a period of 7 days to transmit any additional information) and within 15 days for other EIG I according to the format in force.

A new fact of interest to the research or the product that is the subject of the research is likely to modify the assessment of the benefit/risk ratio and to have an impact on the safety of the subjects taking part in the trial.

These new facts must be notified to the promoter and will be the subject of a declaration to the competent authorities within 15 calendar days following receipt by the promoter.

The sponsor will send a summary table of SAEs I each semester to the CPP and to the trial investigators.

SAEs A will be recorded and kept by the sponsor in order to be declared via the annual safety report.

The protocol, the patient information note and the consent may be amended if new information concerning safety is updated.

### 9.4.3 Independent Oversight Committee

The independent monitoring committee may be contacted at the request of an investigator in the event of a repeat SAE. This committee will be made up of: Dr. Tarik Hasselah (Bichat-Beaujon University Hospital), Prof. Yvon Calmus (Cochin University Hospital), Prof. Pageaux (Saint Eloi Hospital). This committee will be able to analyze the entire medical file of the patient, his technical file concerning the irradiation as well as any treatment document. It will meet every 50 patients included or more depending on the frequency of events.

It can therefore help to make difficult decisions during the trial for which independent judgment is desirable. He may give an opinion in the following circumstances:

- Opinion on premature termination of the trial (for toxicity or because the trial is no longer feasible)
- Opinion on major changes to the protocol that have become necessary due to recruitment or monitoring of the trial, or to take into account new scientific data
- Interim analysis: interpretation of the results of the analysis, request for additional analyzes or data from the trial

The sponsor will send a line listing of adverse effects and events during the committee meeting.

The Independent Committee's opinion is sent in writing to the study sponsor and investigators within 30 days.

### 9.4.4. Annual Safety Report (RAS)

A safety report is produced annually on the anniversary date of the clinical trial authorization (AEC) issued by the competent authority.

This report consists of three parts: report on patient safety, "line-listing" of SAEs and overall summary table.

The report is produced by the research sponsor in collaboration with the coordinating investigator. A period of 60 days (from the anniversary date of the AEC) is given to the promoter to send this document to the competent authorities. As a result, the investigators are required to send the SAEs to the sponsor within the required time frame.

## 9.5. Methods and duration of follow-up of people following the occurrence of adverse events

Any patient included in the protocol, whether he has left the study or is a long-term survivor (beyond the 18th ^month^ after the end of treatment) will be followed up regularly in his referring center like any other patient treated for metastatic colorectal cancer. The follow-up frequency depends on the medical habits of each center.

# 10. Administrative and regulatory aspects

## 10.1. Right of access to data and source documents

The medical data of each patient will only be transmitted to the promoter or any person duly authorized by him, and, if necessary, to the authorized health authorities, under conditions guaranteeing their confidentiality.

The sponsor and the supervisory authorities may request direct access to the medical file for verification of the procedures and/or data of the clinical trial, without breaching confidentiality and within the limits authorized by laws and regulations.

## Trial monitoring – Risk D (100%)

Monitoring will be provided by the Clinical Research Promotion Unit. A CRA will visit each site regularly to carry out quality control of the data reported in the observation books. The CRA ensures that the notebooks contain all the information requested and checks the compliance of the observation notebook with the protocol and the regulations in force.

The observation notebook for each patient must be in line with the source documents (= patient file). The CRA's access to these documents must be facilitated. The ARC is bound by confidentiality with respect to the information it accesses.

The frequency of visits will depend on the number of patients included, the rate of inclusion and the difficulties observed during the study and will be defined by the study sponsor.

On-site monitoring visits will be organized after meeting with the investigator. The CRAs must be able to consult:

- patient data collection notebooks included,
- patient medical and nursing records,
- the investigator workbook.

Basic monitoring will check the following 5 points:

- the presence of signed informed consents,
- compliance with the inclusion criteria,
- the main judgment criterion,
- monitoring and reporting SAEs,
- reporting of new facts.

The protocol was classified according to the level of risk estimated for the patient agreeing to the research. It will be followed as follows.

## Inspection / Audit

As part of the trial, an inspection or audit may take place in one of the investigation centers.

An inspection is an official control carried out by the supervisory authorities in order to assess the admissibility of clinical data, to verify compliance with the legislation and the absence of fraud. Inspectors check documents, logistics, records and any other resources that the authorities consider to be associated with the clinical trial and which may be on the site of the trial itself, at the sponsor's and/or on the premises of the service provider organization (CRO) or in other establishments deemed relevant. An audit is a quality control of the trial; it can be conducted by representatives of the promoter or a company duly mandated by the promoter.

## Ethical considerations

### 10.4.1. Written informed consent

The investigator undertakes to inform the patient in a clear and fair manner of the protocol and to ask him for informed and written consent (information letter and consent form in appendix 4 and 5). He will give the patient a copy of the information leaflet and a consent form. The patient can only be included in the study after having read the information notice and signed and dated the consent form. The investigator must also sign and date the consent form. These two documents will be delivered on paper in 2 copies so that the patient and the investigator can each keep a copy. The investigator's original will be filed in the investigator binder.

### 10.4.2. Committee for the Protection of Persons

The promoter undertakes to submit the study project to the prior authorization of a Committee for the Protection of Persons (CPP). The information communicated concerns, on the one hand, the methods and nature of the research and, on the other hand, the guarantees provided for the patients taking part in this trial.

### Patient anonymity

In order to keep the identities of the patients who participated in the study confidential, the first letter of the surname, the first letter of the first name and the date of birth will be the only information that will appear on the observation book (CRF) and which will make it possible to attach the CRF to the patient a posteriori.

The sponsor is also required to code the patient data on all the documents that he may have in his possession (report of imaging examinations, biology, etc.) which would be attached to the CRF.

### 10.4.4. Computerized data

The computer file used to carry out this research by recording patient data has been the subject of a request for authorization from the CNIL (Commission Nationale Informatique et Libertés), in application of the “Informatique et Libertés” law.

## 10.5. Protocol Amendments

Requests for substantial modifications will be sent by the sponsor for authorization or information to the Afssaps and/or to the committee for the protection of persons concerned in accordance with law 2004-806 of August 9, 2004 and its implementing decrees.

The modified protocol will have to be the subject of a dated updated version.

The patient information and consent forms should be modified if necessary.

## 10.6. Declaration to competent authorities

This protocol will be the subject of an authorization request from the Afssaps.

## 10.7. Financing and insurance

The promoter bears the additional costs related to any supplies or specific examinations required by the research protocol or for its implementation.

This funding plans to cover the following costs:

- promotion (administrative management of the trial, monitoring)
- insurance subscription
- data entry
- data analysis
- Pharmacovigilance
- Medico-economic study
- Examinations considered as an additional cost for the investigation sites
- Traveling patients for study visits

In accordance with article L.1121-10 of chapter I of title II of book I of the first part of the CSP, the sponsor assumes compensation for the harmful consequences of biomedical research for the person who takes part in it and that of his heirs, unless he proves that the damage is not attributable to his fault or that of any party involved, without the fact of a third party or the voluntary withdrawal of the person who had initially consented to lend to research.

The promoter, the Nantes University Hospital *,* declares that it has taken out an insurance policy guaranteeing, according to the clauses provided for in the contract and within the limits of the sums fixed, the pecuniary consequences of its civil liability as it results from the application of the Article L 1121-10 of the Public Health Code. The subscription of such a policy by the promoter does not have the effect of depriving it of its rights of recourse against the aforementioned persons in the event of their fault.

## 10.8. Posting Rules

One year after the end of the study, the final report will be submitted to the promoter who will send a summary to the supervisory authorities.

A copy of the publication will be given to the sponsor of the study who will necessarily be quoted. Apart from the coordinating investigators, the authors will be determined in proportion to the number of patients included.

The protocol has been declared in the American public database: clinicaltrial.gov.

# 11. Feasibility of the project

## 11.1 Workforce

The indications for TACE are at least 50 patients/year per centre, ie more than 500/year. Among these patients, almost half have a possibly irradiable tumor mass. Based on a very low estimate of 1 patient/month/centre over an inclusion period of 24 months, we will be able to include the 175 patients planned, especially since this figure implies 10% of those lost to follow-up. It is clear that certain centers such as Lille, Paul Brousse or Lyon represent particularly significant potential for inclusion.

## 11.2 Recruitment Opportunity

The patients in this study are among the most represented since it is the group of patients with HCC in whom a so-called curative treatment (RFA, resection, TH) is not possible in the short term and can only benefit from a treatment palliative by TACE.

The criteria are broad: all etiologies of cirrhosis, possibility of previous treatments including sorafenib. In the current practice of multidisciplinary oncology consultation meetings, the patients included do not fall under an indication for sorafenib. This could change in the near future because the marketing authorization for sorafenib is intended for patients undergoing palliative treatment, therefore those undergoing TACE therapy. In the current version, any treatment with sorafenib is stopped during the trial. This may be subject to an amendment.

## 11.3 Competition from other tests

Adjuvant therapy trials of TACE ± sorafenib or RFA ± sorafenib are ongoing and will be completed. To our knowledge, there is no randomized trial in Europe on this topic. A similar but non-randomized trial is found in Clinicaltrials.gov.

"Stereotactic Body Radiation Therapy (SBRT) in Combination With Cisplatin Transcatheter Arterial Chemoembolization (TACE) for Primary Hepatocellular Carcinoma (HCC) NCT00746655. University of Pittsburgh."

## 11.4. Trial organization and management

### 11.4.1. Project coordination

- Prof. Cyrille Féray and Prof. Philippe Merle
- Clinical trial coordinator of CIC 04 of Nantes University Hospital (ETP 0.25 x 3 years)
- Conference call / 3 months
- Investigator meetings during the congress of the French Association
- Newsletter every 3 months
- Monitoring ARC (promotion)
- Hepatic CT review committee after the last inclusions
- Pharmacovigilance
- Submission of the trial to Afssaps, CPP and insurance monitoring: Anne Omnes Research Office

### 11.4.2. In each center

- Recruitment within multidisciplinary consultation meetings
- Recruitment within the radiology staff
- Help with recruitment by local TECs remunerated by the PHRC
- Vacations for radiotherapy engineers and technicians

#

# Liste des annexes

[Sommaire 3](#_Toc275873965)

[Annexe 1 : Liste des investigateurs principaux 4](#_Toc275873966)

[Annexe 2 : Composition du comité de surveillance 6](#_Toc275873967)

[Annexe 3 : Synopsis de l’étude 7](#_Toc275873968)

[Annexe 4 : lettre d’information 11](#_Toc275873969)

[Annexe 5 : Formulaire de recueil de consentement des patients 16](#_Toc275873970)

[Annexe 6 : Critères de diagnostique « critères de Barcelone » EASL/AASLD 18](#_Toc275873971)

[Annexe 7 : Critères WHO, RECIST et EASL 19](#_Toc275873972)

[Annexe 8 : Score de Child Pugh 20](#_Toc275873973)

[Annexe 9 : Questionnaire de qualité de vie EORTC-QLQ C30 21](#_Toc275873974)

[Annexe 10 : Echelle d’activité selon ECOG (« Eastern Cooperative Oncology Group ») 22](#_Toc275873975)

[Annexe 11 : Score de morbidité tardive de la radiothérapie RTOG/EORTC 23](#_Toc275873976)

[Annexe 12 : Évaluation de la toxicité aiguë CTC NCI 4.0 24](#_Toc275873977)

[Annexe 13 : Dossier d’information Euro Pharmat – Dispositif médical 25](#_Toc275873978)

[Annexe 14 : CRF suivi de l’étude Annexe 15 : CRF médico-économique 26](#_Toc275873979)

[Annexe 15 : CRF médico-économique 27](#_Toc275873980)

[Annexe 16 : Références du projet 28](#_Toc275873981)

1. Llovet JM, Burroughs A, Bruix J. Hepatocellular carcinoma. Lancet 2003;362:1907-1917

2. Bruix J, Sherman M, Llovet JM, et al. Clinical management of hepatocellular carcinoma. Conclusions of the Barcelona-2000 EASL conference. European Association for the Study of the Liver. J Hepatol 2001;35:421-430

3. Lo CM, Fan ST, Liu CL, et al. Living donor versus deceased donor liver transplantation for early irresectable hepatocellular carcinoma. Br J Surg 2007;94:78-86

4. Yao FY. Expanded criteria for hepatocellular carcinoma: down-staging with a view to liver transplantation--yes. Semin Liver Dis 2006;26:239-247

5. Shiina S, Teratani T, Obi S, et al. A randomized controlled trial of radiofrequency ablation with ethanol injection for small hepatocellular carcinoma. Gastroenterology 2005;129:122-130

6. Llovet JM, Ricci S, Mazzaferro V, et al. Sorafenib in advanced hepatocellular carcinoma. N Engl J Med 2008;359:378-390

7. Cheng AL, Kang YK, Chen Z, et al. Efficacy and safety of sorafenib in patients in the Asia-Pacific region with advanced hepatocellular carcinoma: a phase III randomised, double-blind, placebo-controlled trial. Lancet Oncol 2009;10:25-34

8. Hoffmann K, Glimm H, Radeleff B, et al. Prospective, randomized, double-blind, multi-center, Phase III clinical study on transarterial chemoembolization (TACE) combined with Sorafenib versus TACE plus placebo in patients with hepatocellular cancer before liver transplantation - HeiLivCa [ISRCTN24081794]. BMC Cancer 2008;8:349

9. Llovet JM, Di Bisceglie AM, Bruix J, et al. Design and endpoints of clinical trials in hepatocellular carcinoma. Journal of the National Cancer Institute 2008;100:698-711

10. Lau WY, Leung TW, Ho SK, et al. Adjuvant intra-arterial iodine-131-labelled lipiodol for resectable hepatocellular carcinoma: a prospective randomised trial. Lancet 1999;353:797-801

11. Cheng BQ, Jia CQ, Liu CT, et al. Chemoembolization combined with radiofrequency ablation for patients with hepatocellular carcinoma larger than 3 cm: a randomized controlled trial. Jama 2008;299:1669-1677

12. DeAngelis CD, Fontanarosa PB. Retraction: Cheng B-Q, et al. Chemoembolization combined with radiofrequency ablation for patients with hepatocellular carcinoma larger than 3 cm: a randomized controlled trial. JAMA. 2008;299(14):1669-1677. Jama 2009;301:1931

13. Aaronson NK, Ahmedzai S, Bergman B, et al. The European Organization for Research and Treatment of Cancer QLQ-C30: a quality-of-life instrument for use in international clinical trials in oncology. Journal of the National Cancer Institute 1993;85:365-376

14. Lammer J, Malagari K, Vogl T, et al. Prospective Randomized Study of Doxorubicin-Eluting-Bead Embolization in the Treatment of Hepatocellular Carcinoma: Results of the PRECISION V Study. Cardiovasc Intervent Radiol 2009

15. Lewis AL, Gonzalez MV, Leppard SW, et al. Doxorubicin eluting beads - 1: effects of drug loading on bead characteristics and drug distribution. J Mater Sci Mater Med 2007;18:1691-1699

16. Hong K, Khwaja A, Liapi E, et al. New intra-arterial drug delivery system for the treatment of liver cancer: preclinical assessment in a rabbit model of liver cancer. Clin Cancer Res 2006;12:2563-2567

17. Poon RT, Tso WK, Pang RW, et al. A phase I/II trial of chemoembolization for hepatocellular carcinoma using a novel intra-arterial drug-eluting bead. Clin Gastroenterol Hepatol 2007;5:1100-1108

18. Varela M, Real MI, Burrel M, et al. Chemoembolization of hepatocellular carcinoma with drug eluting beads: efficacy and doxorubicin pharmacokinetics. J Hepatol 2007;46:474-481

19. Dawson LA. The evolving role of radiation therapy in hepatocellular carcinoma. Cancer Radiother 2008;12:96-101

20. Dawson LA, Guha C. Hepatocellular carcinoma: radiation therapy. Cancer J 2008;14:111-116

21. Dawson LA, Ten Haken RK. Partial volume tolerance of the liver to radiation. Semin Radiat Oncol 2005;15:279-283

22. Mornex F, Girard N, Beziat C, et al. Feasibility and efficacy of high-dose three-dimensional-conformal radiotherapy in cirrhotic patients with small-size hepatocellular carcinoma non-eligible for curative therapies--mature results of the French Phase II RTF-1 trial. Int J Radiat Oncol Biol Phys 2006;66:1152-1158

23. Zeng ZC, Tang ZY, Fan J, et al. A comparison of chemoembolization combination with and without radiotherapy for unresectable hepatocellular carcinoma. Cancer J 2004;10:307-316

24. Seong J, Keum KC, Han KH, et al. Combined transcatheter arterial chemoembolization and local radiotherapy of unresectable hepatocellular carcinoma. Int J Radiat Oncol Biol Phys 1999;43:393-397

25. Guo WJ, Yu EX. Evaluation of combined therapy with chemoembolization and irradiation for large hepatocellular carcinoma. Br J Radiol 2000;73:1091-1097

26. Guo WJ, Yu EX, Liu LM, et al. Comparison between chemoembolization combined with radiotherapy and chemoembolization alone for large hepatocellular carcinoma. World J Gastroenterol 2003;9:1697-1701

27. Dawson LA, Eccles C, Craig T. Individualized image guided iso-NTCP based liver cancer SBRT. Acta Oncol 2006;45:856-864

28. Zhou ZH, Liu LM, Chen WW, et al. Combined therapy of transcatheter arterial chemoembolisation and three-dimensional conformal radiotherapy for hepatocellular carcinoma. Br J Radiol 2007;80:194-201

29. Kubas A, Chapet O, Merle P, et al. [Dosimetric impact of breath-hold in the treatment of hepatocellular carcinoma by conformal radiation therapy]. Cancer Radiother 2009;13:24-29

30. Therasse P, Arbuck SG, Eisenhauer EA, et al. New guidelines to evaluate the response to treatment in solid tumors. European Organization for Research and Treatment of Cancer, National Cancer Institute of the United States, National Cancer Institute of Canada. Journal of the National Cancer Institute 2000;92:205-216
