# Supplementary material for: TACE and conformal radiotherapy vs. TACE alone for hepatocellular carcinoma: A randomised controlled trial
Source: JHEP Rep. 2023 Jan 29;5(4):100689. doi: 10.1016/j.jhepr.2023.100689 (PMC10017427; doi:10.1016/j.jhepr.2023.100689)
Supplement: Multimedia component 4 [file mmc4.doc]

**
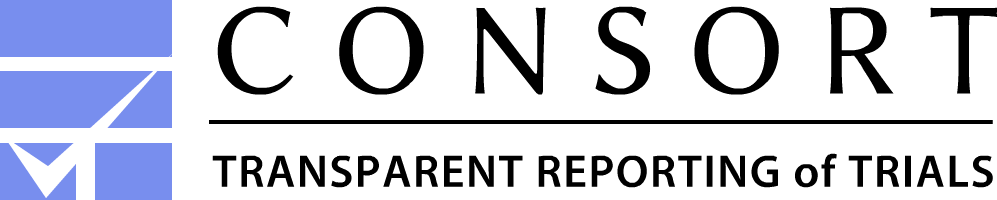
**

**CONSORT 2010 Flow Diagram**

**Allocation**

**Analysis**

**Follow-Up**

**Enrollment**

Assessed for eligibility (n=123 )

Excluded (n=3 )

♦  Not meeting inclusion criteria (n=3 )

♦  Declined to participate (n=0 )

♦  Other reasons (n=0 )

Analysed (n= 55 )
♦ Excluded from analysis (give reasons) (n=0)

Lost to follow-up (give reasons) (n=0)

Discontinued intervention (give reasons) (n= 5, death=2, withdrawal=3 )

Allocated to intervention (n=64 )

♦ Received allocated intervention (n=60 )

♦ Did not receive allocated intervention (give reasons) (n=4 , metastasis=3, withdrawal=1

metastmet

Lost to follow-up (give reasons) (n=0 )

Discontinued intervention (give reasons) (n= 4, death=3, no imagery=1)

Allocated to intervention (n=56 )

♦ Received allocated intervention (n=49 )

♦ Did not receive allocated intervention (give reasons) (n=7 progression=3,CRT or TACE technically impossible= 3, portal thrombosis=1 )

Analysed (n= 45 )
♦ Excluded from analysis (give reasons) (n=0)

Randomized (n=120 )
