# Supplementary material for: TACE and conformal radiotherapy vs. TACE alone for hepatocellular carcinoma: A randomised controlled trial
Source: JHEP Rep. 2023 Jan 29;5(4):100689. doi: 10.1016/j.jhepr.2023.100689 (PMC10017427; doi:10.1016/j.jhepr.2023.100689)
Supplement: Multimedia component 4 [file mmc7.pdf]

TACE and conformal radiotherapy *vs.* TACE alone for hepatocellular carcinoma: A randomised controlled trial

Cyrille Féray,[1,*] Loic Campion,[2] Philippe Mathurin,[3] Isabelle Archambreaud,[4] Xavier Mirabel,[5] Jean Pierre Bronowicki,[6] Emmanuel Rio,[7] Christophe Perret,[8] Laurent Mineur,[9] Frédéric Oberti,[10] Yann Touchefeu,[4] Jérôme Gournay,[4] Hélène Regnault,[11] Julien Edeline,[12] Agnès Rode,[13] Patrick Hillion,[14] Jean Frédéric Blanc,[15] Eric Nguyen Khac,[16] Daniel Azoulay,[1] Alain Luciani,[17] Athena Galetto Preglisasco,[17] Elodie Faurel-Paul,[18] Hélène Auble,[18] Françoise Mornex,[19] Philippe Merle[20]

[1]Centre Hepato-Biliaire, Hôpital Paul Brousse, APHP, Université Paris-Saclay, INSERM 1193, Villejuif France; [2]Department of Biostatistics, Institut de Cancérologie de l'Ouest, Université Nantes, INSERM U307, Nantes, France; [3]Service des Maladies de l'Appareil Digestif, Hôpital Huriez, Université Lille, INSERM 1286, Lille, France; [4]Institut des Maladies de l'Appareil Digestif, Hôtel-Dieu, Nantes, France; [5]Department of Radiation Oncology, Centre Oscar Lambret, Lille, France; [6]Department of Gastroenterology and Hepatology, CHU Nancy-Brabois, Nancy, France; [7]Department of Radiation Oncology, Institut de Cancérologie de l'Ouest, Saint Herblain, France; [8]Radiology Department, Hôtel-Dieu, Nantes, France; [9]Digestive Oncology, Institut Sainte Catherine, Avignon, France; [10]Department of Gastroenterology and Hepatology, Centre Hospitalo-universitaire, Angers, France; [11]Department of Gastroenterology and Hepatology, Hôpital Henri Mondor, APHP; Université Paris Est, Creteil, France; [12]Department of Medical Oncology, Centre Eugène Marquis, Rennes, France; [13]Radiology Department, Hôpital de la Croix-Rousse, Hospice Civil de Lyon; Lyon, France; [14]Department of Gastroenterology and Hepatology, Centre Hospitalo-universitaire, Dijon, France; [15]Department of Gastroenterology and Hepatology, Hôpital Sud Haut-Lévêque, Bordeaux, France; [16]Department of Gastroenterology and Hepatology, Centre Hospitalo-universitaire, Université Amiens, Amiens, France; [17]Radiology Department, Hôpital Henri Mondor, APHP, Créteil, France; [18]Direction de la Recherche Medicale, Hôtel-Dieu, Nantes, France; [19]Department of Radiation Oncology, Centre Hospitalier Lyon Sud, Hospices Civils de Lyon, Université Claude Bernard Lyon, EMR 3738, Lyon, France; [20]Hepatology and Gastroenterology Unit, Hôpital de la Croix-Rousse, Hospices civils de Lyon, Université Claude Bernard, INSERM U1052, Lyon, France

JHEP Reports **2023.** https://doi.org/10.1016/j.jhepr.2023.100689

Background & Aims: Transcatheter arterial chemoembolisation (TACE) is recommended for patients with hepatocellular carcinoma devoid of macrovascular invasion or extrahepatic spread but not eligible for curative therapies. We compared the efficacy and safety of the combination of a single TACE and external conformal radiotherapy (CRT) *vs.* classical TACE.
Methods: TACERTE was an open-labelled, randomised controlled trial with a 1:1 allocation rate to two or three TACE (arm A) or one TACE + CRT (arm B). Participants had a mean age of 70 years, and 86% were male. The aetiology was alcohol in 85%. The primary endpoint was liver progression-free survival (PFS) in the intention-to-treat population. The typical CRT schedule was 54 Gy in 18 sessions of 3 Gy.
Results: Of the 120 participants randomised, 64 were in arm A and 56 in arm B; 100 participants underwent the planned schedule and defined the 'per-protocol' group. In intention-to-treat participants, the liver PFS at 12 and 18 months were 59% and 19% in arm A and 61% and 36% in arm B (hazard ratio [HR] 0.69; 95% CI 0.40–1.18; p = 0.17), respectively. In the per-protocol population, treated liver PFS tended to be better in arm B (HR 0.61; 95% CI 0.34–1.06; p = 0.081) than in arm A. Liver-related grade III–IV adverse events were more frequent in arm B than in arm A. Median overall survival reached 30 months (95% CI 23–35) in arm A and 22 months (95% CI 15.7–26.2) in arm B.
Conclusions: Although TACE + CRT tended to improve local control, this first Western randomised controlled trial showed that the combined strategy failed to increase PFS or overall survival and led more frequently to liver-related adverse effects.
Impact and implications: Hepatocellular carcinoma is frequently treated by arterial embolisation of the tumour and more recently by external radiotherapy. We tried to determine whether combination of the two treatments (irradiation after embolisation) might produce interesting results. Our results in this prospective randomised study were not able to demonstrate a beneficial effect of combining embolisation and irradiation in these patients. On the contrary, we observed more adverse effects with the combined treatment.
Clinical Trials Registration: NCT01300143.
© 2023 The Authors. Published by Elsevier B.V. on behalf of European Association for the Study of the Liver (EASL). This is an open access article under the CC BY license (http://creativecommons.org/licenses/by/4.0/).

Keywords: Hepatocellular carcinoma; Conformal external radiotherapy.
Received 14 October 2022; received in revised form 22 December 2022; accepted 16 January 2023; available online 29 January 2023
* Corresponding author. Address: Centre Hépato Biliaire, Hôpital Paul Brousse. Villejuif, 94800. France. Tel.: +33 1 45596780
E-mail address: Cyrille.feray@aphp.fr (C. Féray).

Introduction

Hepatocellular carcinoma (HCC) is one of the most common cancers worldwide. Most HCC cases are diagnosed at an intermediate or advanced stage, when treatment options such as

surgical resection, liver transplantation, and percutaneous ablation are not appropriate (stages B and C of the Barcelona Clinic Liver Cancer [BCLC] system.[1] Transcatheter arterial chemoembolisation (TACE) is a first-line therapy for patients with BCLC stage B HCC or those with a normal liver function and multinodular tumours without macroscopic vascular invasion or extrahepatic spread. Further, TACE is the treatment of choice for mono- or pauci-nodular HCC that is classified as early HCC but not eligible for curative options.[2]

Combining therapies for HCC is still unusual despite positive results achieved with a combination of TACE and radiofrequency ablation,[3] or TACE and conformal radiotherapy (CRT).[4] The efficacy of TACE with three-dimensional conformal radiotherapy (3-DCRT) for advanced HCC has recently been evaluated but only in Eastern countries, principally in China. Two recent meta-analyses[4,5] reviewed these trials associating TACE and external radiotherapy; they highlighted their methodological weaknesses that resulted in low-certainty evidence, but suggested the superiority of the combination. All the Chinese studies involved HBV or HCV as the major aetiology for liver disease. In a recent review by Haber *et al.*[6] on high-quality randomised clinical trials and HCC, none of them involved the use of external radiotherapy. Little is known about the combination of TACE + CRT in terms of the outcome of patients with early HCC. Here, we carried out a prospective randomised study in and intention-to-treat population to compare the effects on tumour progression of combined therapy (TACE followed by 3-DCRT) *vs.* three TACE procedures, in a Western population of patients with mono- or pauci-nodular HCC who were not eligible for curative options.

Patients and methods

Study design

This was a phase II, open-label, multicentre, prospective, randomised controlled trial. Participants were assigned randomly under a 1:1 design to a single course of TACE + CRT or up to three courses of TACE alone, using a stratified permuted block (n = 4) procedure. The stratification factors were (1) previous curative therapy (yes or no); (2) previous palliative therapy (yes or no); and (3) the administration of sorafenib (yes or no).

Patients volunteered to participate in the study. Written informed consent was obtained from all participants before undergoing any study-specific procedures. All relevant institutional review boards approved the study, which was carried out in accordance with the Declaration of Helsinki and local laws. The study was entered in the US National Institutes of Health Clinical Trials Registry at http://clinicaltrials.gov, and the registration number is NCT01300143. In the TACE-only arm, the participants would undergo up to three sessions of TACE (Weeks 0, 8, and 16), whereas in the TACE + CRT arm, participants underwent a single TACE procedure followed 10–15 days later by external CRT of 54 Gy fractionated into 18 sessions, 5 days per week.

The primary outcome was hepatic progression-free survival (PFS) determined radiologically using modified Response Evaluation Criteria in Solid Tumour (mRECIST). Only patients who met the following inclusion criteria were enrolled in this study: (1) patients aged 18 years and older; (2) Eastern Cooperative Oncology Group (ECOG) performance status 0–1; (3) radiological imaging showing characteristic features of HCC, or one radiological image associated with alpha foetoprotein >400 ng/L, or histological evidence of HCC; (4) maximum lesion size ≤9 cm; (5) not eligible for surgery or percutaneous therapy; (6) Child–Pugh A or B7; (7) aspartate aminotransferase and alanine aminotransferase <7 × the upper limit of normal; (8) conformal external radiotherapy technically possible; (9) TACE technically possible (10) the entire tumour mass could be treated with TACE; and (11) written informed consent signed by the patient. Patients were excluded for the following reasons: (1) metastatic disease; (2) minimum lesion size ≤5 mm; (3) uncontrolled B virus replication; (4) history of radiotherapy at the abdominal level; (5) patients not complying with effective contraception; (6) pregnant or nursing female patients; (7) contraindication of TACE or external conformal radiotherapy; (8) any other concomitant experimental treatment; (9) contraindication of doxorubicin (10) patients unable to comply with respiratory gating procedures if used by the different sites (11) patients unable to understand the information given to them and to follow the protocol instructions; and (12) complete portal vein thrombosis. The trial was halted before the planned number of participants (n = 174) could be included because of participant recruitment problems. This study was funded by the Programme Hospitalier de Recherche Clinique (Hospital Clinical Research Programme) in 2009 (Institut National du Cancer) and was approved by the Tours Ethics Committee (No. 2010-A01089-30).

TACE procedure

TACE was performed according to two different techniques: classical Lipiodol (Lipiodol Ultra-Fluide; André Guerbet Laboratories) or drug-eluting beads (DC Bead; Biocompatibles UK Ltd). For both procedures, portal vein patency and a good blood supply to the liver were confirmed. Under the first method, a mixture of doxorubicin (50 mg; Pharmorubicin; Pfizer) and Lipiodol (5–20 ml) was prepared for TACE. Absorbable Embosphere microspheres (300–500 mm) were used for embolisation. Drug-eluting beads are non-resorbable embolic microspheres that can be loaded with cytotoxic agents. The entire liver tumour burden was treated with TACE during both types of procedure.

In the TACE-only arm, at least two TACE procedures were planned, the second TACE being scheduled at Week 8. A third TACE at Week 16 was indicated depending on the response to the previous TACE.

Conformal radiotherapy

Because the study was carried out in several centres, different systems were used. Gross tumour volume was defined as the tumour volume that was enhanced in the arterial phase and was shown with a washout in the portal venous phase of the computed tomography (CT) scan. The clinical tumour volume (CTV) was generated by adding 5–10 mm to the gross tumour volume. The target volume for planning was expanded to include a 5–10-mm margin from the CTV to compensate for internal physiological movements and variations in the size, shape, and position of the CTV. A respiratory gating technique was used to reduce the dose delivered to healthy tissues and surrounding organs. The typical schedule was 54 Gy delivered over 18 sessions.

The French radiation oncologists involved in this study regularly work together and had previously received detailed guidelines that included volumes, beam orientation preferences, limit doses to adjacent organs, and the total and daily doses to be adapted, and they had participated in specific workshops during national radiotherapy meetings. The radiotherapy regimen was therefore standardised across the sites, but it was too difficult and expensive to implement a quality control programme.

Table 1. Participant characteristics according to therapeutic arm.

Variable	Global	TACE	TACE + CRT	*p*
Sex	120	64	56	0.9721
Male	103 (85.8%)	55 (85.9%)	48 (85.7%)	
Female	17 (14.2%)	9 (14.1%)	8 (14.3%)	
Age (years)	120	64	56	0.1767
Mean ± SD	70.4 ± 9.2	71.5 ± 9.4	69.2 ± 8.8	
Range	(48; 91)	(50; 91)	(48; 89)	
Child–Pugh score	120	64	56	0.5801
A	105 (87.5%)	57 (89.1%)	48 (85.7%)	
B	15 (12.5%)	7 (10.9%)	8 (14.3%)	
ECOG performance status	120	64	56	0.4686
0	73 (60.8%)	37 (57.8%)	36 (64.3%)	
1	47 (39.2%)	27 (42.2%)	20 (35.7%)	
BCLC staging	120	64	56	0.5370
A	89 (74.2%)	46 (71.9%)	43 (76.8%)	
B	26 (21.7%)	14 (21.9%)	12 (21.4%)	
C	5 (4.1%)	4 (6.2%)	1 (1.8%)	
ALBI grade	120	64	56	0.0156
Mean ± SD	-2.46 ± 0.47	-2.56 ± 0.47	-2.35 ± 0.45	
Range	(-3.54; -1.38)	(-3.54; -1.38)	(-3.21; -1.49)	
ALBI grade	120	64	56	0.0260
Grade 1 (≤-2.60)	49 (40.8%)	32 (50.0%)	17 (30.4%)	
Grade 2 (>-2.60/≤1.39)	70 (58.4%)	31 (48.4%)	39 (69.6%)	
Grade 3 (>-1.39)	1 (0.8%)	1 (1.6%)	0 (0.0%)	
Ethnic group	120	64	56	1.0000
Caucasian	116 (96.7%)	62 (96.9%)	54 (96.4%)	
Non-Caucasian	4 (3.3%)	2 (3.1%)	2 (3.6%)	
Cirrhosis	120	64	56	0.0710
No	13 (10.8%)	10 (15.6%)	3 (5.4%)	
Yes	107 (89.2%)	54 (84.4%)	53 (94.6%)	
Cause of liver disease	107	54	53	0.4307
Alcohol	64 (59.8%)	29 (53.7%)	35 (66.0%)	
Hepatitis C	14 (13.1%)	8 (14.8%)	6 (11.3%)	
Hepatitis B	2 (1.9%)	1 (1.9%)	1 (1.9%)	
Alcohol + metabolic	10 (9.3%)	5 (9.3%)	5 (9.4%)	
Alcohol + hepatitis C	2 (1.9%)	2 (3.7%)	0 (0.0%)	
Haemochromatosis + metabolic	1 (0.9%)	0 (0.0%)	1 (1.9%)	
Haemochromatosis	4 (3.7%)	3 (5.6%)	1 (1.9%)	
Autoimmune	1 (0.9%)	0 (0.0%)	1 (1.9%)	
Metabolic	7 (6.5%)	4 (7.4%)	3 (5.7%)	
Other	5 (3.4%)	4 (3.7%)	1 (1.8%)	
Normal liver	10 (8.3%)	8 (12.5%)	2 (3.6%)	
Interval between diagnosis and randomisation (months)	120	64	56	0.5210
Mean ± SD	10.6 ± 18	12.4 ± 21.2	8.5 ± 13.4	
Range	(0.3; 98.7)	(0.3; 98.7)	(0.6; 57)	
Previous therapy	120	64	56	0.5182
None	91 (75.8%)	47 (73.4%)	44 (78.6%)	
Curative	15 (12.5%)	8 (12.5%)	7 (12.5%)	
Curative + TACE	3 (2.5%)	3 (4.7%)	0 (0.0%)	
Palliative	11 (9.2%)	6 (9.4%)	5 (8.9%)	
Previous curative therapy	18	11	7	0.2260
Radiofrequency	13 (72.2%)	9 (81.8%)	3 (42.8%)	
Surgical resection	3 (16.7%)	1 (9.1%)	2 (28.6%)	
Resection + thermoablation	2 (11.1%)	1 (9.1%)	1 (14.3%)	
Previous palliative therapy	14	9	5	
TACE lipiodol	10 (71.5%)	8 (88.9%)	2 (40.0%)	0.1520
TACE DC beads	1 (7.1%)	0 (0.0%)	1 (20.0%)	
TACE lipiodol + DC beads	1 (7.1%)	0 (0.0%)	1 (20.0%)	
TACE lipiodol + sorafenib	1 (7.1%)	1 (11.1%)	0 (0.00%)	
Sorafenib	1 (7.1%)	0 (0.00%)	1 (20.0%)	
No. of lesions from radiology	120	64	56	1.0000
1	78 (65.0%)	41 (64.1%)	37 (66.1%)	
2	31 (25.8%)	17 (26.6%)	14 (25.0%)	
3	8 (6.7%)	4 (6.3%)	4 (7.1%)	
4	2 (1.7%)	1 (1.6%)	1 (1.8%)	
6	1 (0.8%)	1 (1.6%)	0 (0.0%)	
Ascites (radiological)	120	64	56	0.0381
No	108 (90.0%)	61 (95.3%)	47 (83.9%)	
Yes	12 (10.0%)	3 (4.7%)	9 (16.1%)	

(*continued on next page*)

Table 1 (*continued*)

Variable	Global	TACE	TACE + CRT	*p*
Segmental portal invasion (macroscopic)	120	64	56	0.2471
No	117 (97.5%)	61 (95.3%)	56 (100.0%)	
Yes	3 (2.5%)	3 (4.7%)	0 (0.0%)	
Portal hypertension at imaging	120	64	56	0.0209
No	67 (55.8%)	42 (65.6%)	25 (44.6%)	
Yes	53 (44.2%)	22 (34.4%)	31 (55.4%)	
Sum of tumour diameters (mm)	119	63	56	0.7034
Mean ± SD	53.7 ± 23.4	54.2 ± 23.8	53.2 ± 23.2	
Range	(11; 117)	(11; 113)	(17; 117)	
TACE technique	120	64	56	0.8197
DC Beads	91 (75.8%)	48 (75.0%)	43 (76.8%)	
Chimioembolisation lipiodol	29 (24.2%)	16 (25.0%)	13 (23.2%)	
Platelets (/mm^3)	120	64	56	0.9687
Mean ± SD	148.6 ± 75.5	149.3 ± 80.9	147.8 ± 69.6	
Range	(16; 365)	(16; 352)	(45; 365)	
Total bilirubin (mg/L)	120	64	56	0.1116
Mean ± SD	17.4 ± 9.9	16 ± 9.1	18.9 ± 10.7	
Range	(4; 50)	(4; 44.5)	(5; 50)	
Alpha-foetoprotein (ng/ml)	120	64	56	0.088
Low (<200)	100 (83.3%)	57 (89.1%)	43 (76.8%)	
High (≥200)	20 (16.7%)	7 (10.9%)	13 (23.2%)	
International normalised ratio	119	64	55	0.6515
Mean ± SD	1.3 ± 0.4	1.3 ± 0.5	1.2 ± 0.4	
Range	(0.9; 3.4)	(1; 3.4)	(0.9; 3.2)	
Albumin (g/L)	120	64	56	0.0272
Mean ± SD	38.1 ± 4.8	39 ± 4.8	37 ± 4.7	
Range	(26; 49.1)	(28.7; 49.1)	(26; 45)	
Creatinine (µmol/L)	119	64	55	0.3152
Mean ± SD	87.4 ± 60.8	82.2 ± 45.1	93.5 ± 75	
Range	(37.1; 524)	(37.1; 399)	(42; 524)	

Continuous variables are expressed as mean (IQR) and compared using a *t* test. Categorical variables are presented as frequencies (percentages) and compared using Pearson's Chi-square test. ALBI, albumin–bilirubin. BCLC, Barcelona Clinic Liver Cancer; ECOG, Eastern Cooperative Oncology Group; TACE, transcatheter arterial chemoembolisation.

Study endpoints

The primary study endpoint was the liver PFS, defined as the time elapsing between the date of randomisation and that of death or radiological local progression, as determined by mRECIST. For each participant, a maximum of two liver tumours (≥1 cm) were defined as the target lesions, the maximum diameter of the viable tumour being measured. The sample size was computed using the expected radiological liver tumour progression (CT scan) measured by mRECIST. The literature available at that time suggested that up to 60% of patients would experience liver tumour progression within 18 months of TACE.[7] The hypothesis was that, in the ITT and combined arms, there would be a 20% reduction in liver progression at 18 months. Given these elements, in a bilateral situation, with an α risk of 5% and a power of 80%, a planned inclusion period of 24 months, and a minimum follow-up period of 18 months, the number of participants required to obtain 95 events would be N = 75 per arm. Taking account of 15% of participants being lost to follow-up, a total of 174 inclusions was initially planned. Secondary endpoints included time to tumour progression (TTP), overall survival (OS), and per-protocol efficacy and safety. TTP was defined as the time elapsing between randomisation and liver progression. OS was defined as the time elapsing between randomisation and death.

To prevent any incomplete outcome data concerning OS, we used the French national database on specific causes of mortality (CepiDC), which provides dates of death from death registers.

Assessment of tumour response and treatment safety

Treatment response per investigator was assessed initially by contrast-enhanced dynamic CT or magnetic resonance imaging using mRECIST mean measurements of the longest diameter and the sum of nodules.[8] In both arms, tumour response to the entire therapeutic regimen was initially evaluated 4 weeks after each TACE procedure and then every 12 weeks. Participants who did not meet the mRECIST definitions of complete response, partial response, or progressive disease were considered to have stable disease, if evaluable.

The prospective evaluation was not blinded for adverse events (AEs) that were monitored and graded using the National Cancer Institute Common Terminology Criteria for Adverse Events, version 4.0.[9]

Centralised imaging reviews blinded to the therapeutic arm and also to the previous local assessment were assured by expert radiologists (AGP and AL).

Statistical analysis

The first step consisted in a descriptive analysis of the study population. Qualitative factors were described using frequencies of their respective modalities, and continuous factors were described using their mean ± SD. Both treatment strategies (TACE *vs.* TACE + CRT) were compared using Pearson's Chi-square test (or Fisher's test) for qualitative factors and Student's *t* test (or the Mann–Whitney *U* test) for continuous factors.

The primary endpoint was liver PFS determined according to mRECIST. The main criterion was erroneously translated in the ClinicalTrials.gov website as time to progression. The original protocol is submitted as Supplemental information. The progression of treated lesions was also analysed in the per-protocol population. Liver PFS was described using interval-censored Cox estimated curves, and the arms were compared using interval-censored Cox regression. PFS and OS were defined from the

date of randomisation and described using Kaplan–Meier curves. Treatment arms were compared using the classic log-rank test. Time to grade III–IV AE was defined as the interval elapsing between the date of randomisation and that of AE onset (participant was censored at the end of follow-up if no grade III–IV AE occurred).

To maximise the robustness of the results and reduce selection and confounding biases caused by an imbalance in prognostic factors between the two strategies, a propensity score was built using logistic regression with factors (continuous or discretised) linked to the strategy. From the propensity score, a variable was created and calculated according to the 'inverse probability of treatment weighting' method and then introduced into the bivariate model (1 = treatment; 2 = variable derived from the propensity score) for liver PFS, OS, and grade III–IV AE related to portal hypertension.

The strength of the association was estimated using the hazard ratio (HR) or odds ratio, reported with a 95% CI. All statistical analyses were two-tailed, and the significance of p was set at <0.05. The promotor of the trial (Nantes University Hospital) generated the random allocation sequence and organised the centralised reviewing of imaging findings. All data were collected by the clinical research team (EF-P and HA) and transmitted to the study statistician (LC). All analyses were performed using SAS software version 9.4 (SAS institute Inc., Cary, NC, USA) and Stata SE 17.0 (StataCorp LLC, College Station, TX, USA).

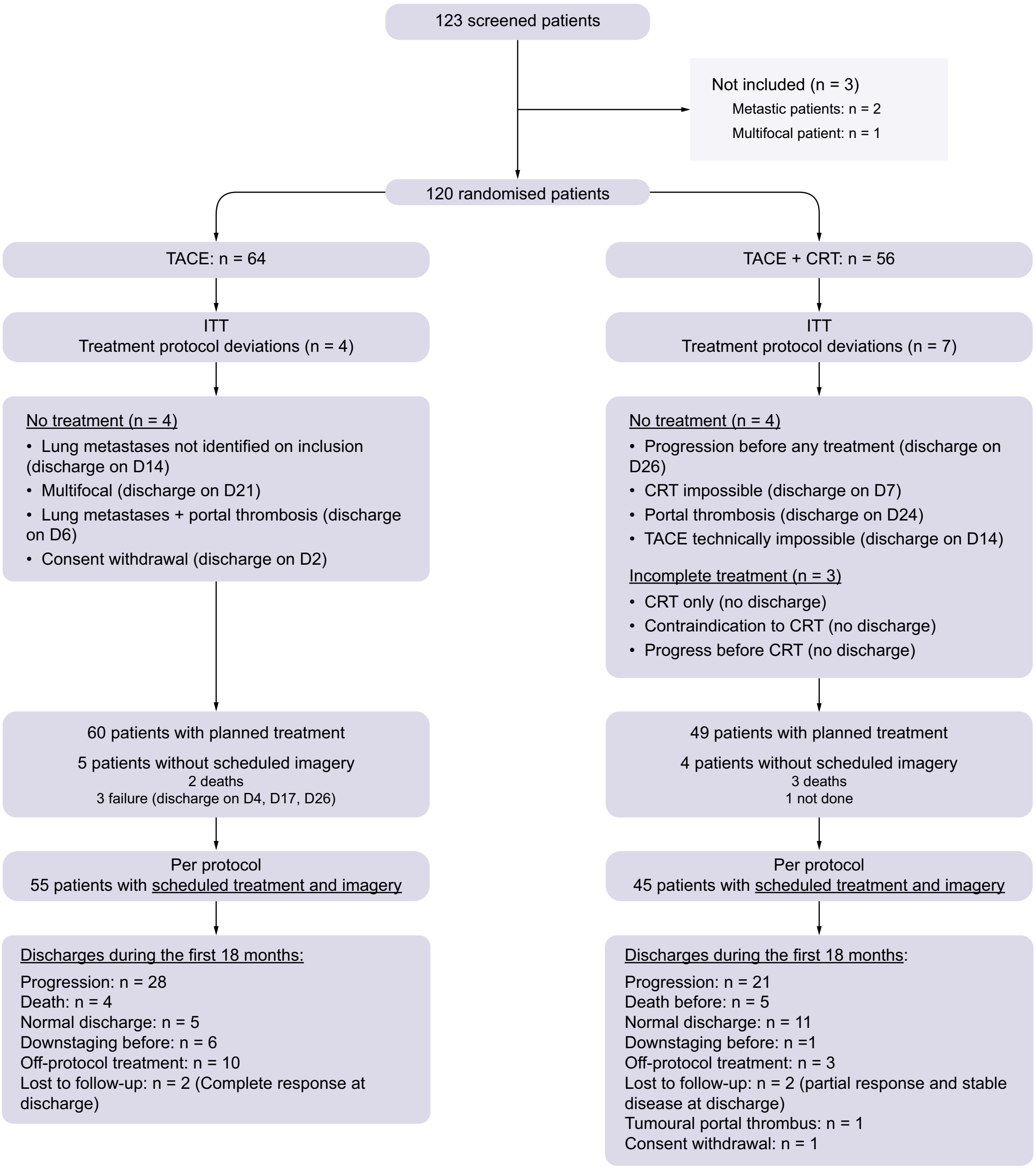

Fig. 1. Flowchart CRT, conformal radiotherapy; ITT, intention-to-treat; TACE, transcatheter arterial chemoembolisation.

Results

Baseline characteristics

Between May 2011 and March 2017, 123 patients with HCC were screened for eligibility in the TACERTE trial, and it was possible to randomise 120 of them (three were affected by exclusion criteria). The minimum follow-up was 36 months. The planned number of 174 participants with a minimum 18 months of follow-up could not be attained. The decision to close the trial was made by the three principal investigators (CF, P Merle, and P Mathurin). The imbalance between the two groups should have been corrected by the randomisation process scheduled for 174 inclusions. For this reason, the predefined statistical endpoints could not be formally tested.

At inclusion, the main characteristics were compared between the two arms (Table 1). Overall, 91/120 (75.8%) participants had not received any previous therapy, 18 had received prior curative therapy (thermoablation in 13 and liver resection in 5), and 13 had undergone a previous TACE procedure. In the combined therapy group, the albumin–bilirubin (ALBI) score, the frequencies of radiological (non-clinical) ascites, and portal hypertension were significantly higher.

Among the 120 randomised participants, 64 were in the TACE-only arm and 56 in the TACE + CRT arm. Twenty participants did not benefit from the planned therapy or follow-up, as shown in Fig. 1. Thus, 100 participants (55 in the TACE-only arm and 45 in the TACE + CRT arm) could be the subject of a per-protocol analysis. The baseline characteristics of treated (per-protocol) participants are given in Table S1.

Because of the incompleteness of the study and the imbalance between the two arms, we also analysed the results using a propensity score.

ITT liver PFS and TTP

It was planned that participants would be monitored radiologically up to Week 72. Median radiological follow-up reached 11 months (range 2–18) among participants who were still alive at Week 72. Cox-calculated liver PFS rates at 6, 12, and 18 months reached 75%, 47%, and 14% in the TACE-only arm and 76%, 47%, and 25% in the TACE + CRT arm, respectively (HR 0.78; 95% CI 0.50–1.20; p = 0.260). Because the two arms differed in terms of serum albumin, bilirubin, and platelet levels; the lymphocyte count; and radiologic signs of portal hypertension, a propensity score was also used to analyse the primary outcome (liver PFS). The difference between the two groups was still not significant (HR 0.63; 95% CI 0.38–1.05; p =0.079) (Fig. 2A).

Cox-calculated liver TTP rates at 6, 12, and 18 months reached 20%, 37%, and 79% in the TACE-only arm and 16%, 37%, and 70% in the TACE + CRT arm, respectively (HR 0.73; 95% CI 0.43–1.25; p =

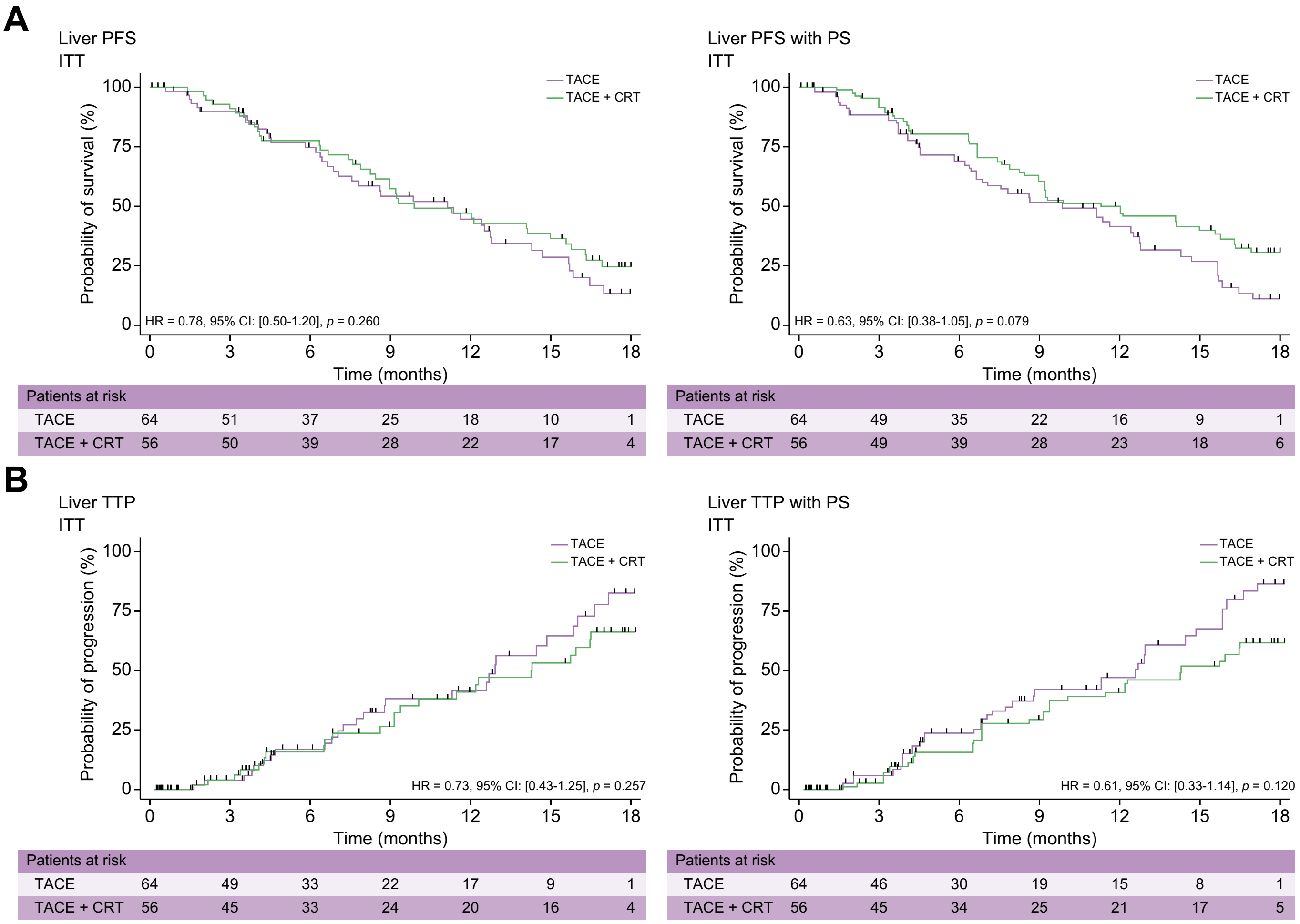

Liver PFS, ITT – Patients at risk

Patients at risk							
TACE	64	51	37	25	18	10	1
TACE + CRT	56	50	39	28	22	17	4

Liver PFS with PS, ITT – Patients at risk

Patients at risk							
TACE	64	49	35	22	16	9	1
TACE + CRT	56	49	39	28	23	18	6

Liver TTP, ITT – Patients at risk

Patients at risk							
TACE	64	49	33	22	17	9	1
TACE + CRT	56	45	33	24	20	16	4

Liver TTP with PS, ITT – Patients at risk

Patients at risk							
TACE	64	46	30	19	15	8	1
TACE + CRT	56	45	34	25	21	17	5

Fig. 2. Kaplan–Meier curves showing (A) liver PFS and (B) liver TTP in the ITT population evaluated without and after PS. CRT, conformal radiotherapy; ITT, intention-to-treat; PFS, progression-free survival; PS, propensity score; TACE, transcatheter arterial chemoembolisation; TTP, time to tumour progression.

0.26). Because the two arms differed in terms of serum albumin, bilirubin, and platelet levels; the lymphocyte count; and radiological signs of portal hypertension, a propensity score was also used to analyse the primary outcome (liver PFS). The difference between the two arms remained not significant (HR 0.61; 95% CI 0.33–1.14; *p* = 0.120) (Fig. 2B).

Per-protocol analysis of treated lesions

Liver PFS rates concerning only the treated lesions at 6, 12, and 18 months reached 83%, 59%, and 19% in the TACE-only arm and 87%, 67%, and 39% in the TACE + CRT arm, respectively (HR 0.64; 95% CI 0.39–1.05; *p* = 0.076). After propensity score (PS), the difference was significant (HR 0.46; 95% CI 0.23–0.89; *p* = 0.021) (Fig. 3A).

Liver TTP rates concerning only the treated lesions at 6, 12, and 18 months reached 19%, 40%, and 78% in the TACE-only arm and 13%, 37%, and 59% in the TACE + CRT arm (HR 0.65; 95% CI 0.37–1.14; *p* = 0.136). After PS, the difference was significant (HR 0.45; 95% CI 0.21–0.94; *p* = 0.033) (Fig. 3B).

Centralised imaging assessment

An independent assessment based on the centralised collection of imaging findings was possible in 96 participants (80%); older imaging records were only available on CD-ROMs and had not been saved on reliable imaging servers. This independent review was blinded with respect to treatment arms. Data were only available for both evaluations in 80 of these 96 participants. Table S2 shows the existence of some discrepancies between the findings of the local and centralised reviews. The Kappa score was 0.38.

From the centralised blinded review available for the 96 participants, the local Cox-calculated liver PFS at 6, 12, and 18 months were 80%, 65%, and 28% in the TACE-only arm and 80%, 63%, and 45% in the TACE + CRT arm, respectively (HR 0.85; 95% CI 0.42–1.75; *p* = 0.66). The results were similar after PS (HR 0.94; 95% CI 0.42–2.13; *p* = 0.88) (Fig. S1).

Safety

Early grade III–IV AEs were observed in 22/56 (39%) participants in the TACE + CRT arm and in 24/64 (38%) participants in the TACE-only arm (*p* = 0.84). Overall numbers of early grade III–IV AEs reached 35 in the TACE + CRT arm and 34 in the TACE-only arm.

Late grade III-IV AEs were observed in 21/56 (38%) participants in the TACE + CRT arm and 14/64 (22%) participants in the TACE-only arm (*p* = 0.060). Overall numbers of late grade III–IV AEs reached 50 in the TACE + CRT arm and 25 in the TACE-only arm.

Late grade III–IV AEs related to portal hypertension (ascites, encephalopathy, or haematemesis) were observed less frequently

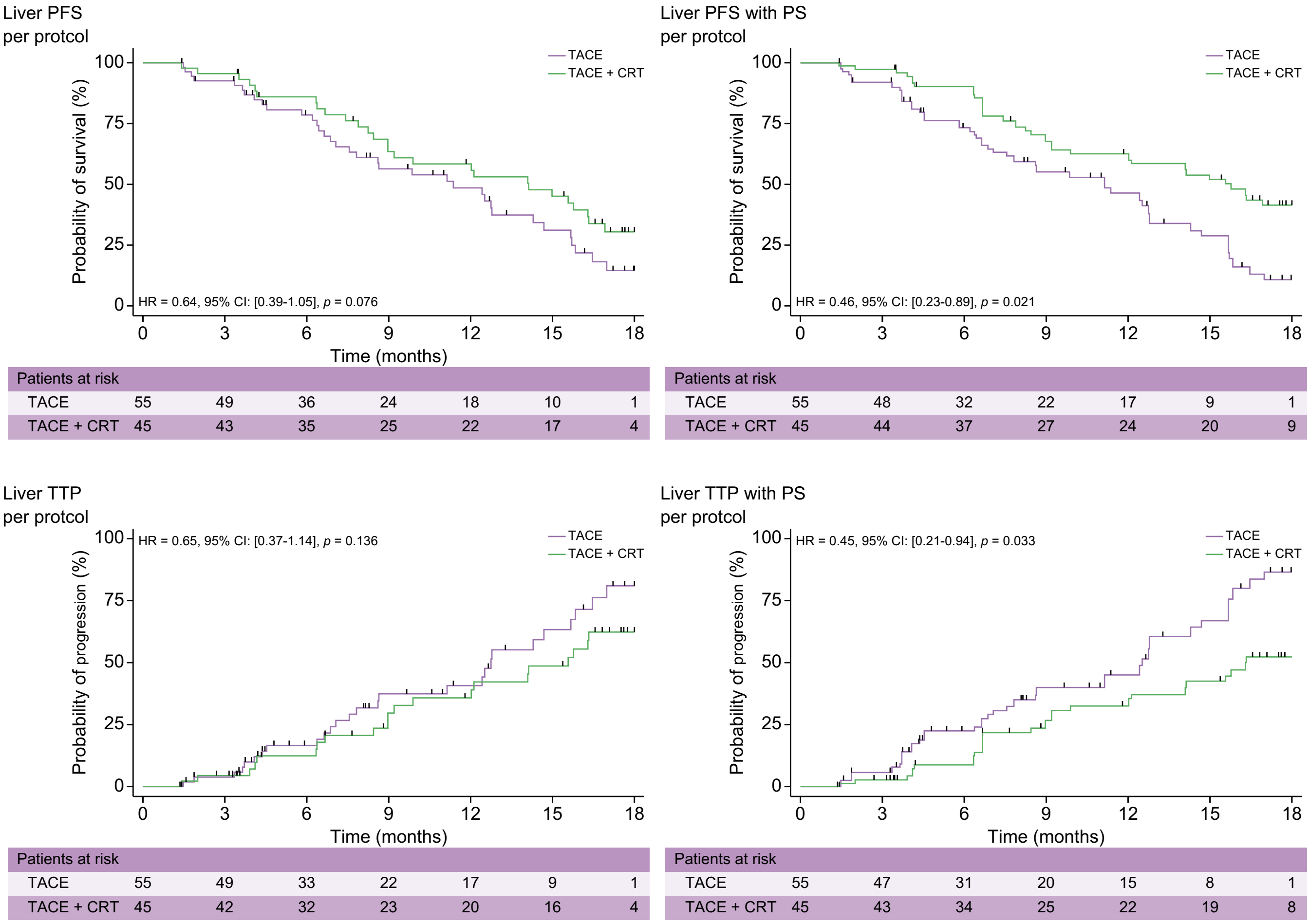

Fig. 3. Kaplan–Meier curves showing treated liver PFS and TTP in the per-protocol population without and after PS. CRT, conformal radiotherapy; ITT, intention-to-treat; PFS, progression-free survival; PS, propensity score; TACE, transcatheter arterial chemoembolisation; TTP, time to tumour progression.

Table 2. Early and late grade III-IV AEs.

Toxicity	AEs before 90 days				Total	AEs after 90 days				Total	
	TACE (n = 35/64)		TACE + CRT (n = 32/56)		Patients	TACE (n = 35/64)		TACE + CRT (n = 32/56)		Patients	
	Patients	Number of AEs	Patients	Number of AEs		Patients	Number of AEs	Patients	Number of AEs		
Cardiac arrest	2	2	0	0	2	0	0	0	0	0	n.s.
Coronary disease	2	2	0	0	2	2	2	1	1	3	n.s.
Cholecystitis	2	2	0	0	2	1	1	0	0	1	n.s.
Cholestasis	6	6	4	4	10	1	2	1	1	2	n.s.
Complication of arteriography	1	1	0	0	1	0	0	0	0	0	n.s.
Cytolysis	4	5	7	10	11	0	0	0	0	0	n.s.
Encephalopathy	0	0	1	1	1	2	2	2	3	4	n.s.
Ascites	**0**	**0**	**1**	**2**	**1**	**2**	**3**	**6**	**6**	**8**	0.012
Haematemesis	0	0	0	0	0	**1**	**1**	**3**	**7**	**4**	<0.01
Portal hypertension-related*	**0**	**0**	**2**	**0**	**2**	**5**	**0**	**11**	**0**	**0**	0.008
General status	**0**	**0**	**4**	**4**	**4**	**1**	**1**	**2**	**3**	**3**	0.05
Arterial hypotension	0	0	1	1	1	0	0	0	0	0	n.s.
Jaundice	1	1	0	0	1	0	0	0	0	0	n.s.
Ionic abnormalities	2	2	0	0	2	1	1	0	0	1	n.s.
Leucopaenia	2	3	6	8	8	1	2	2	2	3	n.s.
Liver failure	2	2	0	0	2	0	0	0	0	0	n.s.
Others	0	0	1	1	1	1	1	4	4	5	<0.01
Pain	1	2	1	1	2	0	0	0	0	0	n.s.
Renal failure	1	1	0	0	1	1	1	2	3	3	n.s.
Sepsis	3	4	3	3	6	2	4	4	4	6	n.s.
Stroke	0	0	1	1	1	0	0	0	0	0	n.s.
Thrombocytopaenia	3	3	1	1	4	0	0	0	0	0	n.s.
Anaemia	0	0	0	0	0	0	0	2	3	2	n.s.
Hand–foot syndrome	0	0	0	0	0	0	0	1	1	1	n.s.
Other cancers	0	0	0	0	0	2	2	1	1	3	n.s.
Pancreatitis	0	0	0	0	0	0	0	1	1	1	n.s.
Upper gastrointestinal	0	0	2	2	2	0	0	4	4	4	n.s.
	32	36	33	39	65	**18**	23	**36**	44	54	

Values in bold denote that AEs significantly more frequent for TACE + CRT.
* Portal hypertension-related AE included ascites, encephalopathy, and haematemesis. AE, adverse event; CRT, conformal radiotherapy; TACE, transcatheter arterial chemoembolisation.

in the TACE-only arm (4/64 [6.3%]) than in the TACE + CRT arm (12/56 [21.4%]) (p = 0.01). During the entire follow-up period for the study, the probability of grade III-IV AEs related to portal hypertension was higher (HR 3.79; 95% CI 1.25–11.48; p = 0.018) in the TACE + CRT arm than in the TACE-only arm. These results were similar after PS (HR 3.81; 95% CI 1.17–12.41; p = 0.02) (Table 2 and Fig. 4).

Overall survival

OS at 12, 24, and 60 months reached 81%, 61%, and 17% in the TACE-only arm and 70%, 46%, and 15% in the TACE + CRT arm, respectively (HR 1.23; 95% CI 0.83–1.82; p = 0.29). The median OS rate reached 30 months (95% CI 23–35) in the TACE-only arm and 22 months (95% CI 15.7–26.2) in the TACE + CRT arm. These results were similar after PS (HR 1.05; 95% CI 0.66–1.70; p = 0.82) (Fig. 5).

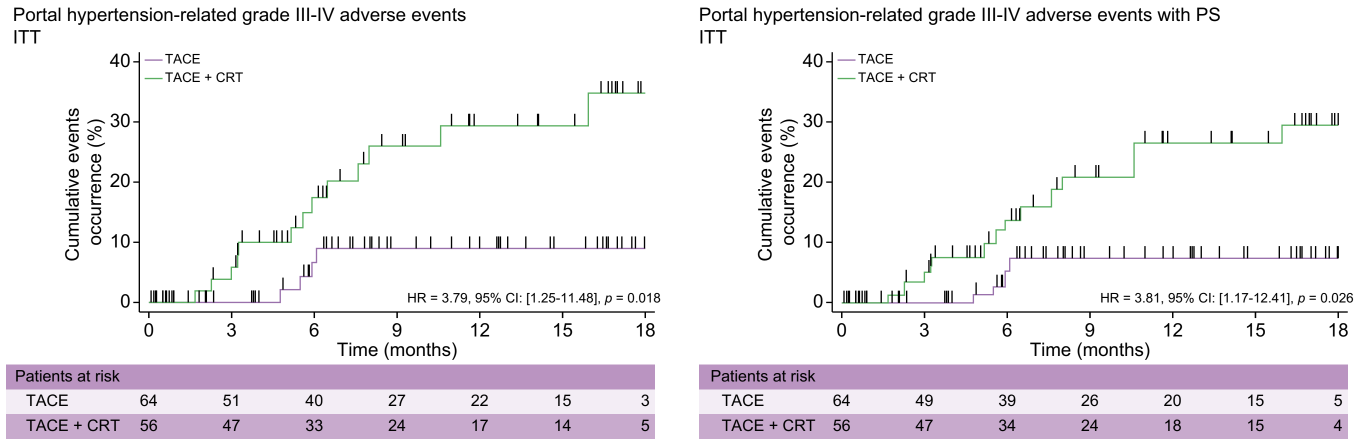

Fig. 4. Kaplan–Meier curves showing probability of portal hypertension-related grade III–IV adverse event in the ITT population without and after PS. CRT, conformal radiotherapy; ITT, intention-to-treat; PS, propensity score; TACE, transcatheter arterial chemoembolisation.

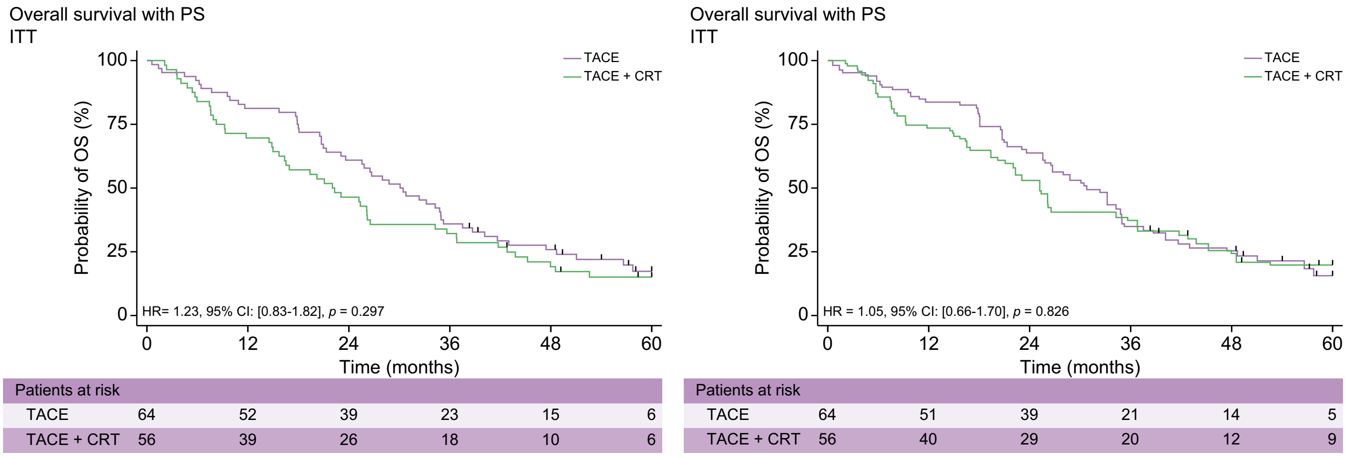

Fig. 5. OS in the ITT population without and after PS. CRT, conformal radiotherapy; ITT, intention-to-treat; OS, overall survival; PS, propensity score; TACE, transcatheter arterial chemoembolisation.

Discussion

This is the first randomised controlled trial to have tested the combination of TACE and CRT for the treatment of mono- or pauci-nodular HCC not eligible for curative options in a Western country. In the Caucasian population thus studied, the primary endpoint was not fulfilled. Furthermore, AEs tended to be more numerous in the combination therapy arm.

Our results contrasted with those of all eight published randomised trials selected by the Cochrane review,[4] which concluded as to a clear efficacy with combination therapy regarding OS and tumour progression. In the present study, the advantages of combined therapy were only observed in the per-protocol population and were only significant after the propensity score was analysed. As in the Chinese trials, most participants tended to have cirrhosis, but the aetiology of liver disease was mostly alcohol in our study, whereas HBV predominated in the Chinese population. The schedule also differed, with just one TACE procedure before CRT, whereas two procedures had been performed during most of the previous trials,[10–16] and even as many as three or five.[17] We used DC Beads in 68% of participants, whereas this was not the case in the Eastern studies. Further, during these studies, CRT was delivered within 4 weeks of the last TACE procedure (as in our study), but the radiation doses received by each individual ranged from 30 to 66 Gy, at rates of 2 to 5 Gy/day and 3 to 5 days per week. Another major difference was that most of the Eastern studies were monocentric and included fewer patients, whereas the present trial was multicentre. One explanation might be that even in small populations, the Chinese centres reporting a striking effect of the combination of TACE and CRT on OS had outstanding expertise in liver embolisation and radiation. A final and more likely hypothesis is that HBV-related HCC is more sensitive to radiotherapy than alcohol-related HCC, which largely predominated in our trial.

Another difference between our trial and the Eastern series was our blinded review of the liver imaging findings for 96/120 participants. This blinded review confirmed the conclusion regarding the absence of a significant impact of combined TACE and CRT on liver PFS. The discrepancies we observed between non-blinded and blinded assessments could be explained by the prospective and multicentre analysis for the present study, whereas the Eastern studies were performed retrospectively and recently by two expert radiologists from a single centre who were blinded to both the therapeutic arm and previous local assessments.

The Eastern trials all reported an absence of any significant AEs in the combination therapy arm, but this was not the case in our study, where the number of grade III–IV liver-related AEs was high in that arm, although we did not benefit from an independent safety committee to determine the relationship between the treatment procedure and the AE. Complications reflective of portal hypertension (ascites, haematemesis, and encephalopathy) tended to be more common in the combination therapy arm and independent of other confounders. This suggests that CRT may cause significant liver toxicity which differs from classic radio-induced liver disease (RILD) (anicteric cholestasis with ascites) or non-classic RILD (marked cytolysis), neither of which were reported during our trial.

The present study had certain limitations. The first was the length of the inclusion period, which enabled the inclusion of only 120 participants, rather than 174. This was at least in part because candidates for both TACE and CRT may be fewer than expected. Moreover, our trial was competing with major clinical trials on yttrium-90 radioembolisation. It should be noted that these trials to test radioembolisation produced negative results with respect to PFS and OS[18,19] and demonstrated additional toxicity in patients treated with internal radiotherapy.[19] Owing to this lack of inclusion, the two groups were unbalanced and thus required propensity score analysis. The other limitation concerned the multicentre nature of our study: the radiological and radiotherapeutic techniques used necessarily differed between centres. Finally, the OS in our trial appeared to be low when compared with that in others. This might have been as a result of the mean age of participants, the frequency of exposure to alcohol and tobacco, the delay between diagnosis and inclusion, and, finally, the degree of liver function, as 60% of the participants were ALBI grade 2.

Although safety was a major concern with CRT, this might logically be less important with stereotaxic body radiation therapy (SBRT), which is more precise than CRT. This technique has emerged during the past decade[20] and is tending to supplant standard CRT. However, the tumour masses in the participants included in our study were relatively high, and around half of them fell outside the current criteria for SBRT (*e.g.* lesion size ≤6 cm or lesion number ≤3, with a total diameter of ≤6 cm).[21] Although SBRT can deliver high doses in a few sessions, it is not certain that SBRT is either more efficient or safer than CRT for the treatment of HCC, and there is a general lack of evidence from randomised trials in this respect.[22]

Abbreviations

3-DCRT, three-dimensional conformal radiotherapy; AE, adverse event; ALBI, albumin–bilirubin; BCLC, Barcelona Clinic Liver Cancer; CRT, conformal radiotherapy; CT, computed tomography; CTV, clinical tumour volume; ECOG, Eastern Cooperative Oncology Group; HCC, hepatocellular carcinoma; HR, hazard ratio; ITT, intention-to-treat; mRECIST, modified Response Evaluation Criteria in Solid Tumour; OS, overall survival; PFS, progression-free survival; RILD, radio-induced liver disease; SBRT, stereotaxic body radiation therapy; TACE, transcatheter arterial chemoembolisation; TTP, time to tumour progression; PS, propensity score.

Financial support

This study was supported by Programme Hospitalier de Recherche Clinique (Hospital Programme for Clinical Research, PHRC) in 2009 (Institut National du Cancer). This study was also supported by PHRC 2010.

Conflicts of interest

CF, LC, IA, XM, ER, CP, LM, FO, YT, JG, HR, JE, AR, PH, JFB, ENK, DA, AGP, EF-P, and HA did not receive any grants or funding. P Merle reports grants or funding from Roche, AstraZeneca, MSD, Eisai, Bayer, and Ipsen. P Mathurin reports grants or funding from Ipsen, Eisai, Abbvie, Sanofi, Gilead Sciences, Pfizer, Evive Biotech, Novo Nordisk, Bayer Healthcare, Surrozen, and Intercept.

Please refer to the accompanying ICMJE disclosure forms for further details.

Authors' contributions

Design of the study: CF, P Merle, FM, JPB, P Mathurin, ER. Recruitment of participants: CF, P Mathurin, IA, JPB, LM, FO, YT, JG, HR, JE, PH, JFB, ENK, DA, P Merle. Radiotherapy: XM, ER, TY, FM. Radiology: CP, AR, AGP, AL. Statistics: LC. Had access to all of the data and can vouch for the integrity of the data analyses: LC. Manuscript authors: CF, LC, P Mathurin, P Merle.

Data availability statement

The data associated with the paper, including the full trial protocol, are available on demand.

Supplementary data

Supplementary data to this article can be found online at https://doi.org/10.1016/j.jhepr.2023.100689.

References

[1] Llovet JM, Brú C, Bruix J. Prognosis of hepatocellular carcinoma: the BCLC staging classification. Semin Liver Dis 1999;19:329–338.
[2] European Association for the Study of the Liver. EASL Clinical Practice Guidelines: management of hepatocellular carcinoma. J Hepatol 2018;69:182–236.
[3] Yang DJ, Luo KL, Liu H, Cai B, Tao GQ, Su XF, et al. Meta-analysis of transcatheter arterial chemoembolization plus radiofrequency ablation versus transcatheter arterial chemoembolization alone for hepatocellular carcinoma. Oncotarget 2017;8:2960–2970.
[4] Lu L, Zeng J, Wen Z, Tang C, Xu N. Transcatheter arterial chemoembolisation followed by three-dimensional conformal radiotherapy versus transcatheter arterial chemoembolisation alone for primary hepatocellular carcinoma in adults. Cochrane Database Syst Rev 2019;2:CD012244.
[5] Huo YR, Eslick GD. Transcatheter arterial chemoembolization plus radiotherapy compared with chemoembolization alone for hepatocellular carcinoma: a systematic review and meta-analysis. JAMA Oncol 2015;1:756–765.
[6] Haber PK, Puigvehí M, Castet F, Lourdusamy V, Montal R, Tabrizian P, et al. Evidence-based management of hepatocellular carcinoma: systematic review and meta-analysis of randomized controlled trials (2002–2020). Gastroenterology 2021;161:879–898.
[7] Lencioni R, Chen XP, Dagher L, Venook AP. Treatment of intermediate/advanced hepatocellular carcinoma in the clinic: how can outcomes be improved? Oncologist 2010;15(Suppl. 4):42–52.
[8] Lencioni R, Llovet JM. Modified RECIST (mRECIST) assessment for hepatocellular carcinoma. Semin Liver Dis 2010;30:52–60.
[9] National Cancer NIH. Institute Common Terminology Criteria for Adverse Events, version 4.0. http://evs.nci.nih.gov/ftp1/CTCAE/CTCAE_4.03_2010-06-14_QuickReference_5 7.pdf.
[10] Zhao MH, Lang FP, Jiang QA, Ma JJ, Song YX. Three-dimensional conformal radiotherapy combined with transcatheter arterial chemoembolization for inoperable primary liver cancer. Chin J Radiat Oncol 2006;15:39–41.
[11] Shang Y, You GX, Xu HY, Chen MC. Prospective randomized clinical study of transcatheter arterial chemoembolization, combined with three-dimensional conformal radiotherapy for primary liver cancer: an analysis of 40 cases. Shijie Xiaohua Zazhi 2007;15:3140–3142.
[12] Xiao ZM, Ouyang TC, Yu RZ, Jiang XS, Ren H, Wu ZJ. Transcatheter arterial chemoembolization combined with 3-dimensional conformal radiotherapy for patients with unresectable primary hepatic carcinoma. Chin J Clin Oncol 2008;35:18–21.
[13] Ning SH, Li GF, Huang YS, Li GS. Three-dimensional conformal radiotherapy combined with transcatheter arterial chemoembolization for hepatocellular carcinoma. Sichuan Med J 2009;30:1896–1898.
[14] Gong QY, Li JG, Kang GJ. Effects of transcatheter arterial chemoembolization (TACE) versus TACE combined with three dimensional radiation (3-DCRT) in the treatment of patients with primary hepatocellular carcinoma. Pract J Cancer 2011;26:634–636.
[15] Xiao P, Dong Y, Sun Y, Liu Q, Cao H, Xu Z, et al. Effect comparison of TACE plus 3DCRT and TACE alone in the treatment of primary hepatic cancer. J Basic Clin Oncol 2011;24:506–507.
[16] Chen WJ, Yuan SF, Zhu LJ, Sun XN, Zheng W. Three-dimensional conformal radiotherapy in combination with transcatheter arterial chemoembolization in the treatment of hepatocellular carcinoma. J BUON 2014;19:692–697.
[17] Liao XF, He HJ, Zhou ZS, Hu W, Zhu XP. Three dimensional conformal radiotherapy combined with interventional therapy for primary hepatocellular carcinoma. J Prac Oncol 2010;25:681–683.
[18] Vilgrain V, Pereira H, Assenat E, Guiu B, Ilonca AD, Pageaux GP, et al. Efficacy and safety of selective internal radiotherapy with yttrium-90 resin microspheres compared with sorafenib in locally advanced and inoperable hepatocellular carcinoma (SARAH): an open-label randomised controlled phase 3 trial. Lancet Oncol 2017;18:1624–1636.
[19] Ricke J, Klümpen HJ, Amthauer H, Bargellini I, Bartenstein P, de Toni EN, et al. Impact of combined selective internal radiation therapy and sorafenib on survival in advanced hepatocellular carcinoma. J Hepatol 2019;71:1164–1174.
[20] Riou O, Azria D, Mornex F. [Stereotactic body radiotherapy for liver tumors: state of the art]. Cancer Radiother 2017;21:563–573.
[21] Durand-Labrunie J, Baumann AS, Ayav A, Laurent V, Boleslawski E, Cattan S, et al. Curative irradiation treatment of hepatocellular carcinoma: a multicenter phase 2 trial. Int J Radiat Oncol Biol Phys 2020;107:116–125.
[22] Chen LC, Chiou WY, Lin HY, Lee MS, Lo YC, Huang LW, et al. Comparing stereotactic ablative radiotherapy (SABR) versus re-trans-catheter arterial chemoembolization (re-TACE) for hepatocellular carcinoma patients who had incomplete response after initial TACE (TASABR): a randomized controlled trial. BMC Cancer 2019;19:275.

Journal of Hepatology, Volume *5*

Supplemental information

TACE and conformal radiotherapy *vs*. TACE alone for hepatocellular carcinoma: A randomised controlled trial

Cyrille Féray, Loic Campion, Philippe Mathurin, Isabelle Archambreaud, Xavier Mirabel, Jean Pierre Bronowicki, Emmanuel Rio, Christophe Perret, Laurent Mineur, Frédéric Oberti, Yann Touchefeu, Jérôme Gournay, Hélène Regnault, Julien Edeline, Agnès Rode, Patrick Hillion, Jean Frédéric Blanc, Eric Nguyen Khac, Daniel Azoulay, Alain Luciani, Athena Galetto Preglisasco, Elodie Faurel-Paul, Hélène Auble, Françoise Mornex, and Philippe Merle

TACE and conformal radiotherapy *vs*. TACE alone for hepatocellular carcinoma: a randomised controlled trial

Cyrille Féray, Loic Campion, Philippe Mathurin, Isabelle Archambreaud, Xavier Mirabel, Jean Pierre Bronowicki, Emmanuel Rio, Christophe Perret, Laurent Mineur, Frederic Oberti, Yann Touchefeu, Jérôme Gournay, Hélène Regnault, Julien Edeline, Agnès Rode, Patrick Hillion, Jean Fréderic Blanc, Eric Nguyen Khac, Daniel Azoulay, Alain Luciani, Athena Galetto Preglisasco, Elodie Faurel-Paul, Hélène Auble, Françoise Mornex, Philippe Merle

Table of content

Fig. S1...2

Table S1..3

Table S2..6

Fig. S1. Liver PFS in 96 patients with centralized review of imaging blinded to therapeutical arm.

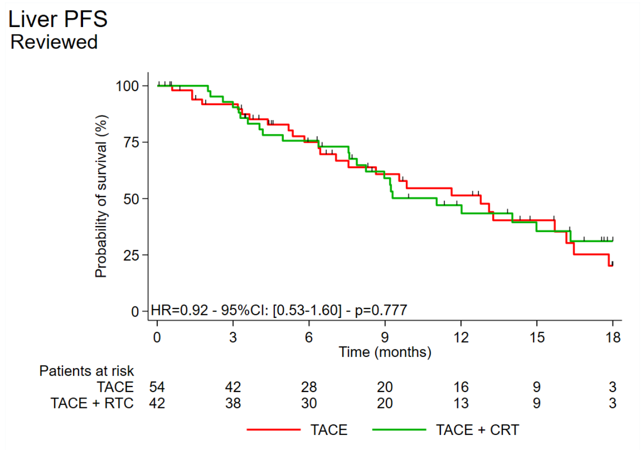

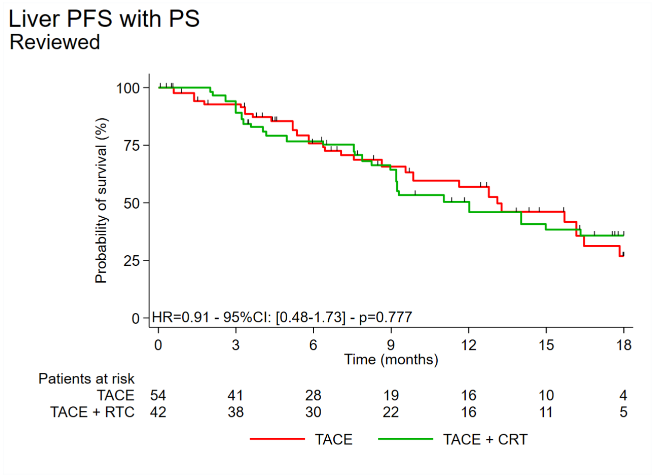

Table S1. Per-protocol patients characteristics

Variable	Global	TACE	TACE+RTC	*p*
**Gender**	100	55	45	*p = 0.8881*
Male	85 (85.0%)	47 (85.5%)	38 (84.4%)	
Female	15 (15.0%)	8 (14.5%)	7 (15.6%)	
**Age (years)**	100	55	45	*p = 0.1132*
Mean ± SD	70.7 ± 9.5	72.1 ± 9.6	69 ± 9.2	
Range	(48 ; 91)	(50 ; 91)	(48 ; 89)	
**Child-Pugh Score**	100	55	45	*p = 0.4919*
A	87 (87.0%)	49 (89.1%)	38 (84.4%)	
B	13 (13.0%)	6 (10.9%)	7 (15.6%)	
**ECOG**	100	55	45	*p = 0.1435*
ECOG 0	61 (61.0%)	30 (54.5%)	31 (68.9%)	
ECOG 1	39 (39.0%)	25 (45.5%)	14 (31.1%)	
**BCLC**	100	55	45	*p = 0.8984*
A	76 (76.0%)	42 (76.4%)	34 (75.6%)	
B	23 (23.0%)	12 (21.8%)	11 (24.4%)	
C	1 (1.0%)	1 (1.8%)	0 (0.0%)	
**ALBI Score**	100	55	45	*p = 0.0603*
Mean ± SD	-2.5 ± 0.5	-2.6 ± 0.5	-2.4 ± 0.4	
Range	(-3.5 ; -1.4)	(-3.5 ; -1.4)	(-3.2 ; -1.5)	
**ALBI Grade**	100	55	45	*p = 0.0421*
Grade 1	42 (42.0%)	28 (50.9%)	14 (31.1%)	
Grade 2	57 (57.0%)	26 (47.3%)	31 (68.9%)	
Grade 3	1 (1.0%)	1 (1.8%)	0 (0.0%)	
**Ethnic group**	100	55	45	*p = 0.5867*
Caucasian	97 (97.0%)	54 (98.2%)	43 (95.6%)	
Non-Caucasian	3 (3.0%)	1 (1.8%)	2 (4.4%)	
**Cirrhosis**	100	55	45	*p = 0.0212*
No	10 (10.0%)	9 (16.4%)	1 (2.2%)	
Yes	90 (90.0%)	46 (83.6%)	44 (97.8%)	
**Cause of liver disease**	100	55	45	*p = 0.2376*
Alcohol	52 (52.0%)	25 (45.5%)	27 (60.0%)	
Hepatitis C	10 (10.0%)	5 (9.1%)	5 (11.1%)	
Hepatitis B	2 (2.0%)	1 (1.8%)	1 (2.2%)	
Alcohol+Metabolic	10 (10.0%)	5 (9.1%)	5 (11.1%)	
Alcohol+Hepatitis C	2 (2.0%)	2 (3.6%)	0 (0.0%)	
Hemochromatosis+Metabolic	1 (1.0%)	0 (0.0%)	1 (2.2%)	
Hemochromatosis	4 (4.0%)	3 (5.5%)	1 (2.2%)	
Autoimmune	1 (1.0%)	0 (0.0%)	1 (2.2%)	
Metabolic	7 (7.0%)	4 (7.3%)	3 (6.7%)	
Other	1 (1.0%)	1 (1.8%)	0 (0.0%)	
Normal liver	10 (10.0%)	9 (16.4%)	1 (2.2%)	
**Diagnostic CHC**	100	55	45	*p = 0.7960*
Radiology	68 (68.0%)	38 (69.1%)	30 (66.7%)	
Biopsy	32 (32.0%)	17 (30.9%)	15 (33.3%)	
**Interval between diagnosis and randomization (months)**	100	55	45	*p = 0.2528*
Mean ± SD	10.8 ± 18.4	12.7 ± 21.9	8.4 ± 12.8	
Range	(0.3 ; 98.7)	(0.3 ; 98.7)	(0.6 ; 49.4)	
**Previous therapy**	100	55	45	*p = 0.5870*
None	74 (74.0%)	39 (70.9%)	35 (77.8%)	

Curative	14 (14.0%)	8 (14.5%)	6 (13.3%)	
Curative+TACE	3 (3.0%)	3 (5.5%)	0 (0.0%)	
Palliative	9 (9.0%)	5 (9.1%)	4 (8.9%)	
**Previous curative therapy**	17	11	6	*p = 0.1250*
Radiofrequency	11 (64.7%)	9 (81.8%)	2 (42.8%)	
Surgical Resection	3 (17.6%)	1 (9.1%)	2 (28.6%)	
Resection+Thermoablation	2 (11.8%)	1 (9.1%)	1 (14.3%)	
Alcoholization	1 (5.9%)	0 (0.0%)	1 (14.3%)	
**Previous palliative therapy**	12	8	4	*p = 0.0670*
TACE Lipiodol	8 (66.8%)	7 (87.5%)	1 (25.0%)	
TACE DC beads	1 (8.3%)	0 (0.0%)	1 (25.0%)	
TACE lipiodol+DC beads	1 (8.3%)	0 (0.0%)	1 (25.0%)	
TACE lipiodol+Sorafenib	1 (8.3%)	1 (12.5%)	0 (0.00%)	
Sorafenib	1 (8.3%)	0 (0.00%)	1 (25.0%)	
**Sorafenid**	100	55	45	*p = 1.0000*
No	98 (98.0%)	54 (98.2%)	44 (97.8%)	
Yes	2 (2.0%)	1 (1.8%)	1 (2.2%)	
**No. of lesions from radiology**	100	55	45	*p = 0.9536*
1	65 (65.0%)	36 (65.5%)	29 (64.4%)	
2	26 (26.0%)	14 (25.5%)	12 (26.7%)	
3	7 (7.0%)	4 (7.3%)	3 (6.7%)	
4	1 (1.0%)	0 (0.0%)	1 (2.2%)	
6	1 (1.0%)	1 (1.8%)	0 (0.0%)	
**Ascites (radiological)**	100	55	45	*p = 0.0743*
No	91 (91.0%)	53 (96.4%)	38 (84.4%)	
Yes	9 (9.0%)	2 (3.6%)	7 (15.6%)	
**Segmental Portal invasion (macroscopic)**	100	55	45	*p = 1.0000*
No	99 (99.0%)	54 (98.2%)	45 (100.0%)	
Yes	1 (1.0%)	1 (1.8%)	0 (0.0%)	
**Sum of tumor diameters (mm)**	99	54	45	*p = 0.8482*
Mean ± SD	54.3 ± 23.5	53.9 ± 23.1	54.8 ± 24.2	
Range	(11 ; 117)	(11 ; 113)	(17 ; 117)	
**TACE technique**	100	55	45	*p = 0.5617*
DC-Beads	75 (75.0%)	40 (72.7%)	35 (77.8%)	
Chimioembolisation lipiodol	25 (25.0%)	15 (27.3%)	10 (22.2%)	
**Hemoglobin (g/L)**	97	54	43	*p = 0.6970*
Mean ± SD	13.5 ± 1.7	13.4 ± 1.9	13.5 ± 1.5	
Range	(6 ; 17)	(6 ; 16.5)	(10.8 ; 17)	
**Leukocytes (103/mm3)**	97	54	43	*p = 0.8744*
Mean ± SD	5.8 ± 2.1	5.8 ± 2.2	5.8 ± 2.1	
Range	(2 ; 11.4)	(2 ; 10.3)	(2.2 ; 11.4)	
**Neutrophils (103/mm3)**	97	54	43	*p = 0.3204*
Mean ± SD	3.6 ± 1.6	3.8 ± 1.7	3.5 ± 1.5	
Range	(1.1 ; 8.4)	(1.2 ; 7.7)	(1.1 ; 8.4)	
**Lymphocytes (/mm3)**	100	55	45	*p = 0.2421*
Mean ± SD	1380.4 ± 648.2	1311.5 ± 556.3	1464.6 ± 743.2	
Range	(270 ; 4220)	(270 ; 3500)	(530 ; 4220)	
**Platelets (103/mm3)**	100	55	45	*p = 0.8633*
Mean ± SD	145.8 ± 76.8	144.5 ± 79.2	147.2 ± 74.5	
Range	(16 ; 365)	(16 ; 352)	(45 ; 365)	
**ASAT (IU/l)**	100	55	45	*p = 0.6255*
Mean ± SD	47.5 ± 28.1	46.2 ± 27.2	49 ± 29.3	
Range	(17 ; 160.8)	(18 ; 132.6)	(17 ; 160.8)	
**ALAT (IU/l)**	100	55	45	*p = 0.7005*
Mean ± SD	37.8 ± 28.5	36.8 ± 23.4	39.1 ± 33.9	
Range	(11 ; 208.2)	(11 ; 147)	(13.8 ; 208.2)	
**Alkaline Phosphatases (IU/l)**	99	55	44	*p = 0.7770*
Mean ± SD	125.4 ± 101.1	122.9 ± 90.5	128.7 ± 114	

Range	(56 ; 832)	(56 ; 693)	(62 ; 832)	
**Gamma-GT (IU/l)**	99	55	44	*p = 0.4498*
Mean ± SD	254.6 ± 260.6	236.8 ± 213.2	276.9 ± 311.1	
Range	(21 ; 1539)	(21 ; 1108)	(37 1539)	
**Total bilirubin (mG/L)**	100	55	45	*p = 0.2574*
Mean ± SD	17.2 ± 9.8	16.2 ± 9.1	18.4 ± 10.6	
Range	(4 ; 50)	(4 ; 44.5)	(5 ; 50)	
**Alfa FP (ng/mL)**	100	55	45	*p = 0.1638*
Mean ± SD	179.8 ± 635.8	99.5 ± 321.6	277.9 ± 874.4	
Range	(0.3 ; 4290)	(0.5 ; 1960.6)	(0.3 ; 4290)	
**Alfa FP (ng/mL)**	100	55	45	*p = 0.1178*
Low (<200)	86 (86.0%)	50 (90.9%)	36 (80.0%)	
High (>=200)	14 (14.0%)	5 (9.1%)	9 (20.0%)	
**INR**	100	55	45	*p = 0.9060*
Mean ± SD	1.2 ± 0.4	1.3 ± 0.5	1.2 ± 0.4	
Range	(0.9 ; 3.3)	(1 ; 3.3)	(0.9 ; 3.2)	
**Albumin (G/L)**	100	55	45	*p = 0.0842*
Mean ± SD	38.2 ± 4.8	39 ± 4.8	37.3 ± 4.7	
Range	(26 ; 49.1)	(28.7 ; 49.1)	(26 ; 45)	
**Creatinine (µmol/L)**	99	55	44	*p = 0.6251*
Mean ± SD	87 ± 59.7	84.3 ± 47.6	90.3 ± 72.4	
Range	(42 ; 524)	(44.3 ; 399)	(42 ; 524)	
**Sodium (mmol/L)**	98	54	44	*p = 0.5537*
Mean ± SD	138.9 ± 2.9	138.7 ± 3.1	139 ± 2.7	
Range	(128 ; 145)	(128 ; 145)	(131 ; 145)	
**Lymphocytes (/mm3)**	100	55	45	*p = 0.3223*
>=800	88 (88.0%)	50 (90.9%)	38 (84.4%)	
<800	12 (12.0%)	5 (9.1%)	7 (15.6%)	
**Platelets (/mm3)**	100	55	45	*p = 0.0214*
>=75000	87 (87.0%)	44 (80.0%)	43 (95.6%)	
<75000	13 (13.0%)	11 (20.0%)	2 (4.4%)	
**Total bilirubin (mg/L)**	100	55	45	*p = 0.1913*
<20	71 (71.0%)	42 (76.4%)	29 (64.4%)	
>=20	29 (29.0%)	13 (23.6%)	16 (35.6%)	

Table S2

Concordance between blinded and non-blinded review

	Non-blinded review					
Blinded revi	Censored	Stable disease	Progression	Complete response	Partial response	Total
Complete response	1	0	6	10	1	18
Partial resp	0	5	9	1	8	23
Stable disea	1	2	3	0	5	11
Progression	0	5	23	0	2	30
Censored	12	0	0	2	0	14
Total	14	12	41	13	16	96

Among the 96 patients with both evaluations, 16 were censored due to a very short follow-up period (only evaluation at inclusion available).

For the 80 patients for whom both evaluations were available:

	Non-blinded review		
Blinded revi	No progression	Progression	Total
No progressi	32	18	50
Progression	7	23	30
Total	39	41	80

Agreement = 68.75%/Expected agreement=49.69%/**Kappa=0.3789** (p=0.0002)
